# Pulp and paper mill sludges decrease soil erodibility

**DOI:** 10.1002/jeq2.20170

**Published:** 2020-12-08

**Authors:** Kimmo Rasa, Taina Pennanen, Krista Peltoniemi, Sannakajsa Velmala, Hannu Fritze, Janne Kaseva, Juuso Joona, Risto Uusitalo

**Affiliations:** ^1^ Natural Resources Institute Finland Tietotie 4 Jokioinen FI‐31600 Finland; ^2^ Natural Resources Institute Finland Latokartanonkaari 9 Helsinki FI‐00790 Finland; ^3^ Soilfood Oy Viikinkaari 6 Helsinki FI‐00790 Finland

## Abstract

Declining carbon (C) content in agricultural soils threatens soil fertility and makes soil prone to erosion, which could be rectified with organic soil amendments. In a 4‐yr field trial, we made a single application of three different organic sludges from the pulp and paper industry and studied their effects on cereal yield, soil C content, and fungal and bacterial composition. In laboratory rainfall simulations, we also studied the effects of the soil amendments on susceptibility to erosion and nutrient mobilization of a clay‐textured soil by measuring the quality of percolation water passing through 40‐cm intact soil monoliths during 2‐d rainfall simulations over four consecutive years after application. A nutrient‐poor fiber sludge reduced wheat yield in the first growing season, but there were no other significant effects on cereal yield or grain quality. An input of ∼8 Mg ha^−1^ C with the soil amendments had only minor effects on soil C content after 4 yr, likely because of fast microbe‐mediated turnover. The amendments clearly changed the fungal and bacterial community composition. All amendments significantly reduced suspended solids (SS) and total phosphorus (TP) concentrations in percolation water. The effect declined with time, but the reduction in SS and TP was still >25% 4 yr after application. We attributed the lower tendency for particle detachment in rain simulations to direct interactions of soil minerals with the added particulate organic matter and microbe‐derived compounds that stabilize soil aggregates. In soils with low organic matter content, pulp and paper industry by‐products can be a viable measure for erosion mitigation.

**Abbreviations**
CPMS: composted pulp mill sludge
DOC: dissolved organic carbon
DRP: dissolved reactive phosphorus
EC: electrical conductivity
FS: fiber sludge
LPMS: lime‐stabilized pulp mill sludge
PP: particulate phosphorus
SS: suspended solids
TP: total phosphorus.

## 1 INTRODUCTION

The pulp and paper industry produces large quantities of organic sludges as side streams (in Finland, 420,000 Mg dry matter per year) that are incinerated with low net energy recovery (Joona, Kuisma, Alakukku, & Kahiluoto, 2012). In a circular economy, these valuable organic side streams could be used elsewhere in new value chains, creating jobs, business opportunities, and environmental benefits. Agricultural use of organic sludges derived from the pulp and paper industry has been studied for different soil types and climates, with the results generally indicating positive impacts on crop production and soil physical properties (e.g., Chow, Rees, Fahmy, & Monteith, 2003; Gallardo, Cea, Tortella, & Diez, 2012; Sippola, Mäkelä‐Kurtto, & Rantala, 2003). For example, Chow, Rees, Fahmy, and Monteith (2003) reported that pulp mill fibers improved soil aggregation on a gravelly loam soil at application rates of 20–160 Mg ha^−1^. They also found that it took longer before surface runoff started and that soil loss was reduced up to 90% from the level of control when pulp fiber was used as soil amendment. However, less is known about whether these organic side streams can contribute to water protection (i.e., reduced nutrient transport) in clayey agricultural soils for a longer period by improving soil structure and protecting the soil surface against water erosion over several years.

In general, organic matter in soils enhances aggregate stability (Greenland, Lindstrom, & Quirk, 1962; Soinne, Hyväluoma, Ketoja, & Turtola, 2016; Tisdall & Oades, 1982). Also, organic soil amendments can directly form organo‐mineral compounds that bind soil particles together. At the same time, mineralization of introduced organic matter is likely to cause changes in microbial activity and community composition, which affects soil structural properties (Abdi et al., 2017; Gallardo et al., 2012; Zibilske, Clapham, & Rourke, 2000). Microbiological decomposition changes the chemical composition of the added organic matter and provides new types of compounds (e.g., extracellular polymeric substances, fungal hyphae, and microbial necromass), contributing to soil aggregation (Costa, Raaijmakers, & Kuramae, 2018; Peele & Beale, 1941). Decomposition of relatively slowly degradable organic compounds, such as cellulose and lignin present in pulp and paper industry sludges, provides a platform for a succession of microbial substances potentially forming organo‐mineral associations with clay‐sized soil particles. If introduced organic matter increases the degree of soil aggregation and aggregate strength, the greater aggregate stability makes the soil less prone to erosion (Diacono & Montemurro, 2010; Le Bissonnais, 1996).

The carbon (C) content of agricultural soils is currently declining in Finland (Heikkinen, Ketoja, Nuutinen, & Regina, 2013) and on a global scale (Lal, 2004). Organic soil amendments are an attractive option to increase and stabilize soil C storage in the long term (Poulton, Johnston, Macdonald, White, & Powlson, 2018; Six, Conant, Paul, & Paustian, 2002). Organic side streams from the pulp and paper industry have been shown to decompose rather quickly (Foley & Cooperband, 2002; Zibilske et al., 2000). Thus, the effect of a single addition of organic matter on soil structure and C content is likely transient. However, recent literature highlights the importance of microbially derived organic material in stabilizing C via organo‐mineral interactions and protecting it against decomposition (Kopittke et al., 2018, 2020). Carbon protected by organo‐mineral associations leads to the formation of small, water‐stable micro‐aggregates, contributing to soil structural stability over several years (Tisdall & Oades, 1982). In theory, enhanced microbial activity and potential changes in microbial communities due to added organic matter can contribute simultaneously to long‐term C storage and protection of soil structure against water‐induced stresses.

The effect of pulp mill sludge on soil structure and the role of soil microbiology have been reported by Bipfubusa, Angers, N'Dayegamiye, and Antoun (2008). They measured increased aggregate stability after 2 yr of pulp mill sludge application (fresh and composted) on loam‐textured soil containing 20% clay. They suggested that both stimulation of soil microflora and binding of humic substances with soil mineral particles contributed to aggregate stabilization. Abdi et al. (2017) observed changes in microbial community structure for 3 yr after 9 yr of continuous application of pulp mill biosolids, whereas no changes were observed when soil was amended with liming materials.

**Core Ideas**

- Erosion mitigation using pulp and paper mill sludges was tested in a 4‐yr study.
- Intact soil monoliths were taken from field for laboratory rainfall simulations.
- Sludge addition reduced particle and P losses from soil to percolation water.
- Sludges decomposed quickly and had minor effects on soil C content after 4 yr.
- Sludge addition clearly altered soil bacterial and fungal community composition.

In a 4‐yr field experiment, we examined the potential of three different pulp and paper industry organic sludges to reduce the susceptibility of a clay‐textured soil to erosion and nutrient mobilization (rainfall simulation test), the potential to increase soil organic matter content (soil sampling), and the potential to bring about changes in soil microbiology (DNA sequencing). Our starting hypotheses were that (a) organic matter input derived from pulp and paper industry side streams can improve soil structural stability, which reduces soil dispersion during rain events and the risk of associated off‐site nutrient transfer through structured clay soils, and (b) the effect will vary depending on soil amendment properties and over time. We also tested the hypotheses that (c) a single, large input of organic soil amendments will preserve soil organic C over several years and (d) application of wood‐derived amendments to an agricultural soil is reflected in soil microbial activity and community structure. Although research on the functionality of the soil microbial community and its responses in terms of soil functions is limited (Nannipieri et al., 2003; Yang et al., 2018), these interactions are increasingly highlighted when promoting sustainable management of soils and attempts to maintain soil C stocks (Liang, Amelung, Lehmann, & Kästner, 2019). In the present study, we attempted to comprehend changes in erosion vulnerability due to pulp mill sludge amendments using a combination of research methods dealing with soil physical, chemical, and microbiological properties.

## 2 MATERIALS AND METHODS

### 2.1 Origin and quality of soil amendments

The three soil amendments used in the experiment were produced by Soilfood Oy (formerly Tyynelän maanparannus Oy) from organic side streams provided by Stora Enso's Imatra Mill located in southeastern Finland. These were (a) composted pulp mill sludge (CPMS) and (b) lime‐stabilized pulp mill sludge (LPMS), both derived from the process water treatment plant at the factory, and (c) fiber sludge (FS), which consists of cellulose fibers that are too short for the final product of the mill. The main difference between the materials is that those recovered from the mill's wastewater treatment process contain phosphorus (P), nitrogen (N), and other nutrients added to the biological purification step to cut down oxygen demand of effluent waters, whereas FS is a nutrient‐poor cellulose material from the pre‐clarifier of cardboard machine process water, removed in a wire sieve as semi‐dry mass. The origin, processing, analytical methods, and detailed properties of the soil amendments derived from these side streams are given in the Supplemental Material (p. S2, Supplemental Table S1).

### 2.2 Field experiment

The field experiment was established at Jokioinen in southwestern Finland on a clay‐textured soil classified as a Luvic Stagnosol (Eutric, Clayic, Protovertic) (IUSS Working Group, 2015).

The experiment layout was a randomized complete block design with five replicates with a total of 20 plots, each measuring 6 by 15 m. Unamended plots served as the control treatment. The amendment rates used for the sludges at their original moisture content were 52, 51, and 72 Mg ha^−1^ for LPMS, CPMS, and FS, respectively (Table 1). These rates were based on an attempt to increase soil C content as much as possible without exceeding the soluble N (Sol‐N) limit of 30 kg ha^−1^ allowed as autumn application. The sludges were spread on the soil in September 2015 as a single application in the beginning of the experiment, and the field was tilled immediately to a depth of approximately 10 cm. Details of establishment and maintenance of the experiment are given in the Supplemental Material (pp. S2–S4, Supplemental Table S2).

**TABLE 1:** Dry matter content (DM) of the three pulp and paper mill side streams used as soil amendments and amount of organic matter (OM), C, total N (TN), soluble N (Sol‐N), and total P, K, S, Ca, and Mg supplied to soil with the amendments

Sludge^a^	DM	OM	C	TN	Sol‐N	P	K	S	Ca	Cd	Cr
	%	Mg ha^−1^		kg ha^−1^						g ha^−1^	
CPMS	43.4	14.3	7.8	211	34	45	39	109	949	21	727
LPMS	49.7	17.3	9.0	253	32	53	30	131	2,181	16	528
FS	33.5	15.7	8.4	13	1	2	1	7	2,269	0.2	100

^a^ CPMS, composted sludge; FS, fiber sludge; LPMS, lime‐stabilized sludge.

All field plots were sampled at the 0‐to‐20‐cm depth in autumn 2015, just before the amendments were applied, and again in autumn 2019, by taking from each plot three replicate samples that were combined for analyses. At the initial sampling in 2015, soil clay content, determined by a pipette method (Elonen, 1971), was 47%. Total C and total N (LECO CN‐2000) content was 2.3 and 0.19%, respectively. Soil test P (1‐h extraction at 1:10 vol/vol ammonium acetate at pH 4.65; Vuorinen & Mäkitie, 1955) was 10 mg L^−l^, which indicates that annual P fertilization would be unlikely to give yield responses (Valkama, Uusitalo, Ylivainio, Virkajärvi, & Turtola, 2009). Soil pH (H_2_O) was 6.4, and electrical conductivity (EC) was 0.051 μS cm^−1^.

Total C, total N, pH, and EC were determined for samples taken from each field plot at the end of the experiment (autumn 2019). Additionally, soil total cadmium (Cd) content in samples from each plot was analyzed by using a graphite furnace atomic absorption spectrofotometer (AA280Z, Varian) in aqua regia digestate (SFS‐ISO 11466:2007).

In 2016 and 2019, the crop grown was wheat (*Triticum aestivum* L.); in 2017 and 2018, the crop was oats (*Avena sativa* L.). At the end of each growing season, the plots were harvested with an experimental harvester, fresh weight of crop biomass was recorded, and grain samples were dried at 105 °C overnight to calculate moisture content. For analyses of nutrient and heavy metal content, grain samples were milled with a hammer mill and digested with 7 M HNO_3_, and the extracts were analyzed using inductively coupled plasma‐optical emission spectrometry.

### 2.3 Rainfall simulation test

To investigate the ability of the soil amendments to stabilize soil aggregates, and thus reduce erosion and nutrient leaching through soil profile, large undisturbed soil monoliths (30 cm in diameter, ∼40 cm deep) were extracted from all field plots in sections of polyvinyl chloride sewage pipe using a tractor‐driven soil auger. Sampling was repeated in four consecutive springs (May 2016–May 2019).

After coring and transport to the laboratory, the bottom of the monoliths was prepared to expose intact natural ped surfaces. Void spaces created during preparation were filled with washed 3‐to‐5‐mm quartz sand. On the top side of each polyvinyl chloride cylinder, a 32‐mm‐diameter hole was drilled (center of the drill hole at the level of soil surface) to lead water out of the monolith surface if ponding occurs. The monoliths were then saturated from below for 1 d, maintained at saturation for an additional 2 d, and allowed to drain overnight. The monolith sampling and rainfall simulation procedures are described in detail in Uusitalo et al. (2012).

Simulated rain was applied for 5 h d^−1^ on two consecutive days at an intensity of 5 mm h^−1^ (25 mm in both days). Four individual percolation water samples were collected from each monolith during the rainfall simulation. Samples were analyzed separately to detect possible within‐simulation trends. The first water sample was taken from water draining after the saturation period, the second and fourth samples were taken during the consecutive simulated rainfall events, and the third sample was taken from water draining during the night between the events. The total volume of percolation water was close to equal in all treatments, and typically all rain applied percolated through the soil (Supplemental Figure S1). All samples were immediately analyzed for turbidity (2100 AN IS Turbidimeter, Hach), and subsamples were passed through a 0.2‐μm filter (Nuclepore, Whatman). All unfiltered samples and filtered subsamples were frozen and stored at −18 °C for later analysis.

Dissolved reactive P (DRP) was analyzed in the filtered (0.2 μm) subsamples (Lachat QuikChem Method 10‐115‐01‐1‐Q), and total P (TP) was analyzed after acid peroxodisulfate digestion of unfiltered samples in an autoclave (120 °C, 100 kPa, 30 min; Turtola, 1996) based on molybdate colorimetry (Murphy & Riley, 1962). Particulate P (PP) was taken as the difference between TP and DRP. Water samples were further analyzed for suspended solids (SS) (material retained on a Whatman GF/A 1.6‐μm filter), dissolved organic C (DOC) and total organic C (Shimadzu TOC‐V CSH Total organic C analyzer), pH, EC (electrodes Mettler Toledo InLab Expert Pro‐ISM and InLab 731‐ISM, respectively), and total N (unfiltered), nitrate‐N (NO_3_–N), ammonium‐N (NH_4_–N; Lachat QuikChem Methods 10‐107‐04‐2‐C, 10‐107‐04‐2‐C and 10‐107‐06‐2‐B, respectively), calcium (Ca^2+^), potassium (K^+^), magnesium (Mg^2+^), sodium (Na^+^), sulfur (S), and Cd using inductively coupled plasma‐optical emission spectrometry.

### 2.4 Microbiological analyses

Samples for microbial analyses were taken from all 20 field plots (0‐to‐10‐cm depth, composite samples comprising 10 subsamples) in spring and autumn 2018, 3 yr after the single application of the soil amendments. The methods used to analyze basal respiration, microbial biomass (amount of C and N in microbial biomass), phospholipid fatty acids, and soil‐extractable C and N are presented in the Supplemental Material (pp. S5–S6). The methods used for DNA extraction, amplicon sequencing, and bioinformatics are presented briefly below. Glomalin‐related soil proteins were analyzed in triplicate 0.25‐g samples of air‐dry soil (autumn samples only) as described in Moragues‐Saitua, Merino‐Martín, Stokes, and Staunton (2019).

The DNA in both spring and autumn samples from all 20 plots was extracted using the NucleoSpin soil kit (Macherey) and sequenced at the Institute of Genomics, Tartu University, as paired‐end 2 × 300 bp with the MiSeq platform (Illumina) using the MiSeq v3 kit, producing about 20–25 million reads per flow cell. For bacteria, the 16S SSU rRNA gene V4 region was amplified using primers 515F and 806R (Caporaso et al., 2011, 2012). For fungi, the ribosomal internal transcribed spacer 2 region was amplified using primers gITS7 (Ihrmark et al., 2012) and ITS4 (White, Bruns, Lee, & Taylor, 1990) with 8 bp dual index for 24 cycles. Raw sequences of 40 samples have been stored in the NCBI genebank BioProject PRJNA607883 under accession numbers SAMN14150014‐53.

Quality filtering, removal of artefacts, chimeric sequences, primer‐dimers, and primers from raw 16S rRNA and internal transcribed spacer 2 sequence reads and clustering and taxonomy annotation were conducted using the PipeCraft 1.0 pipeline (Anslan, Bahram, Hiiesalu, & Tedersoo, 2017) as described in Soinne et al. (2020). Details are provided in the Supplemental Material (pp. S5 and S6).

### 2.5 Statistical analyses

Preliminary inspection showed that there were no clear within‐simulation trends in SS or nutrient concentrations, so the results for the four individual water samples taken from each monolith were pooled for statistical analysis. The statistical analyses were performed using generalized linear mixed models. Treatment (control, FS, LPMS, CPMS) and year (2016–2019) and their interaction were used as fixed effects, and block and the interaction of block × year were used as random effects. Correlated observations between years were taken into account using the most suitable covariance structure (homogeneous or heterogeneous compound symmetry or first‐order autoregressive, or unstructured). The unstructured covariance matrix is the most flexible because it imposes no pattern on the covariances, whereas CS assumes a constant covariance between all years. The lowest Akaike information criterion value was used as the most important criterion for selection of covariance structure, together with normality of the residuals (Gbur et al., 2012).

Microbial variables (basal respiration, microbial C and N, extractable C and N, glomalin‐related soil protein, and phospholipid fatty acids) were analyzed with the same model using two correlated seasons (spring and autumn) instead of years. Glomalin‐related soil proteins were determined only for autumn samples. Unequal variances of treatments were allowed for total phospholipid fatty acids based on a lower Akaike information criterion value and a likelihood ratio test.

Parameters measured in soil samples (C, N, Cd, EC, and pH) were analyzed from one depth (0–20 cm), using only treatment as a fixed effect and block as a random effect.

Assumptions of gamma (with log link for PP, TP, DRP, and NH_4_–N) and Gaussian (with identity link for the other parameters analyzed) distributions were used for all dependent variables. All models were fitted by using the residual pseudo likelihood (for gamma) or the restricted maximum likelihood (for Gaussian) estimation method. The method of Westfall (1997) was used for pairwise comparisons of treatments with the control within the same year, depth, or season. Pairwise comparison of treatment effect over years was conducted with the Tukey–Kramer method. A significance level of α = .05 was used in all analyses. Degrees of freedom were calculated using the Kenward–Roger method (Kenward & Roger, 2009). The analyses were performed using the GLIMMIX procedure in the SAS Enterprise Guide 7.15 (SAS Institute).

Operational taxonomic unit data from amplicon sequencing were normalized using the GMPR method (Chen, Reeve, Zhang, Huang Wang, & Chen, 2018) in R 3.5.2. One outlier sample, LPMS autumn, was removed because of its small library size. Permutational multivariate ANOVA was performed using distance matrices with function adonis from vegan 2.5–5 (Oksanen et al., 2019), with block as the stratum. Nonmetric multidimensional scaling was conducted with stable solution from random starts, axis scaling, and species scores with function metaMDS from vegan using the Bray–Curtis dissimilarity index and plotted with fitted environmental variables envfit from vegan.

## 3 RESULTS

### 3.1 Rainfall simulation study

**FIGURE 1:**
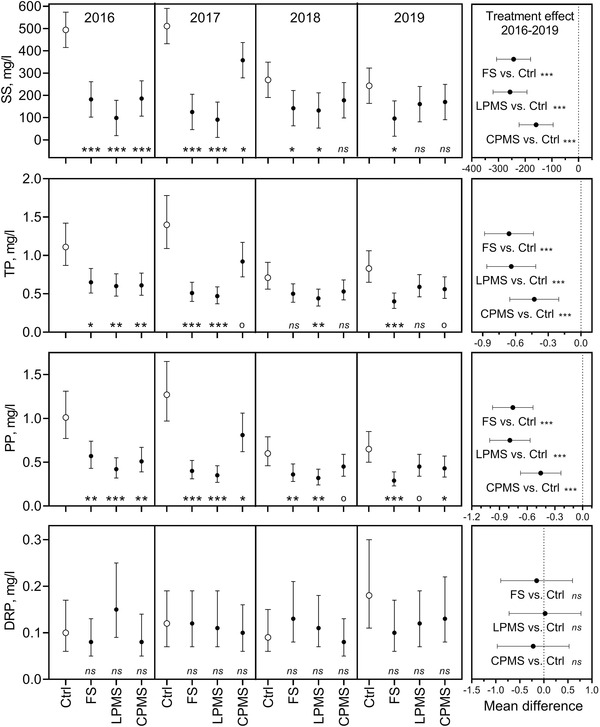
Concentrations of suspended solids (SS), total phosphorus (TP), particulate P (PP), and dissolved reactive P (DRP) in percolation water from 40‐cm deep monoliths of control and treated soil. Treatment effects over 4 yr are summarized in the right‐hand panels, where the differences of means are represented in a link scale when the dependent variable was not normally distributed. Error bars denote 95% confidence intervals. Asterisks indicate significant difference between treatment and control (^*^ *p *< .05, ^**^ *p *< .01, ^***^ *p *< .001, ^O^ *p *< .1; ns [*p* ≥ .1]). Ctrl, control; CPMS, composted sludge; FS, fiber sludge; LPMS, lime‐stabilized sludge

Rainfall simulations carried out over four consecutive years after application of the different pulp and paper industry side streams suggested that all amendments tested significantly decreased soil susceptibility to particle mobilization and associated PP losses (Figure 1; Supplemental Table S3). In all individual years, the unamended control produced the highest SS, PP, and TP concentrations in percolation water (Figure 1), although differences between the control and treatments varied between years. In contrast, mobilization of DRP, which made up 11–33% of TP in percolation water, was not affected by application of the different organic amendments (Figure 1) despite the fact that they all slightly increased soil pH (Supplementary Material Table S4), which could have increased P solubility.

Particle mobilization in amended soil was suppressed more strongly during the first 2 yr of the study, when particle concentration in percolation water of the control treatment was twice as high (∼500 mg L^−1^), than in the latter 2 yr (mean SS, ∼250 mg L^−1^). Whether the effect of the amendments was gradually subsiding with time or if the less pronounced effect of the amendments was due to more stable soil structure in the drier conditions in the latter half of the study period remains unresolved.

Of the three organic soil amendments studied, FS appeared to act most consistently throughout the years, with a mean 47–76% reduction in SS compared with the control. The lowest mean reduction in SS mobilization was associated with CPMS, but it still resulted in a 62% reduction in SS concentration compared with the control in the first year and a 30–34% reduction during the last 3 yr. The highest single‐year SS reduction (80–82%) was associated with LPMS in the first 2 yr; thereafter, the effect was 30–51% compared with the control. Mobilization and transport of PP with percolation water closely followed the same trends as SS because of the natural close correlation between these parameters.

**FIGURE 2:**
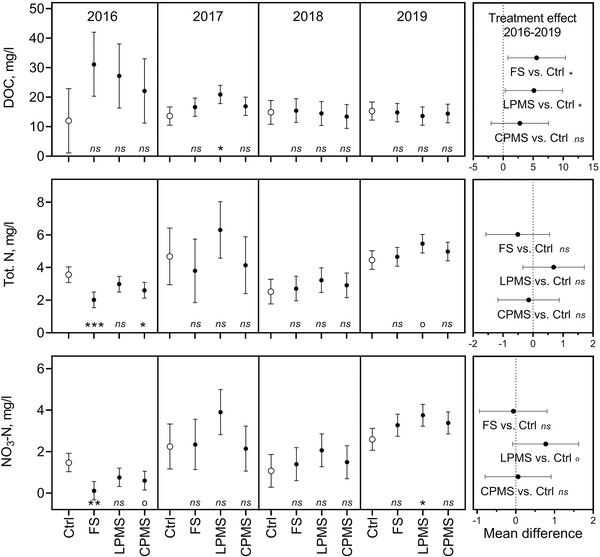
Concentrations of dissolved organic C (DOC), total N, and NO_3_–N in percolation water from 40‐cm‐deep monoliths of control and treated soil monoliths. Treatment effects over 4 yr are summarized in the right‐hand panels. Error bars denote 95% confidence intervals. Asterisks indicate significance difference between treatment and control (^*^ *p *< .05, ^**^ *p *< .01, ^***^ *p *< .005, ^O^ *p *< .1; ns [*p ≥* .1]). For fiber sludge (FS) treatment year 2017, data on total N and NO_3_–N of one of the five replicate samples were removed as outliers. Ctrl, control; CPMS, composted sludge; LPMS, lime‐stabilized sludge

Overall, the FS and LPMS treatments resulted in significant increases in DOC mobilization (Figure 2). A flush of DOC was recorded in percolation water collected during the first year's rainfall simulation (7 mo after amendment application) in all treatments, with 84–160% higher concentrations for the amended soils compared with the control. However, the water samples from the control treatment had highly variable DOC in the first year, and no statistically significant differences were established. In the second study year, DOC concentrations in percolation water were significantly higher in the LPMS treatment (by 54%), whereas DOC concentrations in the other treatments were similar to that in the control. No differences between treatments and control were observed during the last 2 yr of the study (for inorganic and total C, see Supplemental Table S5).

Total N and NO_3_–N concentrations tended to be lower in amended soils than in the control in the first study year but were typically similar or higher in the later years (Figure 2). The overall treatment effects regarding N species in percolation water were not significantly different from the control (Figure 2). The pH and EC of percolation water increased in FS and LPMS treatments, whereas no effect was observed in CPMS treatment (Supplemental Figure S2). For other elements in percolation water, see Supplemental Table S6.

### 3.2 Effect on cereal yield

No significant treatment effects on yield or yield quality were observed during the study (Supplemental Figure S3; Supplemental Tables S7 and S8). However, in the first study year, the grain yield was 14% lower in the nutrient‐poor FS treatment compared with the control. This was likely due to immobilization of N. In the second year, yield in the LPMS treatment was over 500 kg ha^−1^ higher than in the control treatment (*p* = .2). Due to very dry spring in the third year, the mean yields were exceptionally low (<1,500 kg ha^−1^).

### 3.3 Heavy metals

One of the main concerns related to agricultural use of industrial side streams is whether they contain heavy metals or other harmful elements (Cd in particular) that may accumulate in the soil and end up in plants. Of the soil amendments studied here, FS did not contain quantifiable amounts of Cd, whereas the Cd content in CPMS and LPMS was 0.96 and 0.60 mg kg^−1^ DM, respectively (Supplemental Table S1). These concentrations where unexpectedly high, but they did not exceed the maximum permissible Cd concentration for soil amendments under Finnish legislation, which is 1.5 mg kg^−1^ DM. Due to high application rates (>20 Mg ha^−1^ DM), the total Cd loads were 20 and 14 g ha^−1^ for CPMS and LPMS, respectively. For these soil amendments, application exceeded the maximum permissible Cd load of 7.5 g ha^−1^ summed for a 5‐yr period. However, soil Cd concentration did not change due to Cd application with the amendments (Supplemental Table S4). Moreover, Cd and lead (Pb) concentrations in grain did not exceed the maximum permissible levels (0.1 and 0.2 mg kg^−1^ wet weight, respectively) set by European legislation (European Union, 2006; Supplemental Tables S7 and S8). Water samples from the rainfall experiment were also analyzed for Cd in the third and fourth years of the study, but all values were below the detection limit of 0.7 μg L^−1^ (data not shown). Concentrations of other harmful elements were low enough to allow even higher amendment rates than used in this study (Supplemental Table S1).

### 3.4 Effects of amendments on soil and microbes

Four years after application of a single large dose of the organic soil amendments, only minor effects on soil C and N content were detectable (Supplemental Table S4), apart from the CPMS treatment having slightly higher (*p *= .053) soil C content than the control (by 0.18 percentage points).

**FIGURE 3:**
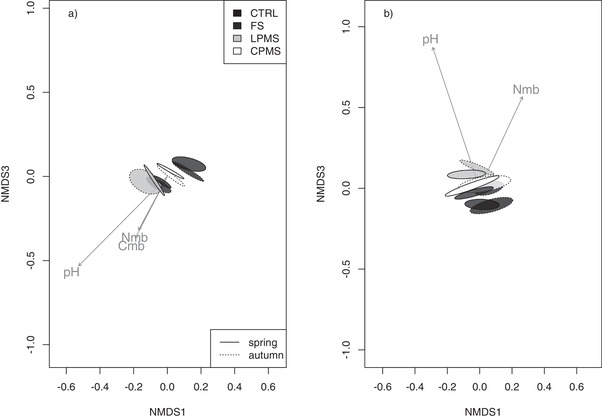
Plot of (a) bacterial and (b) fungal communities in control and treated soil, shown as 95% confidence interval ellipses of SD of the mean of five replicate samples (three‐dimensional nonmetric multidimensional scaling [NMDS] with Bray–Curtis dissimilarity index, showing only axis 1 and axis 3). Grey arrows show increasing direction of significantly correlated (*p *< .01) environmental factors pH, microbial C (Cmb), and microbial N (Nmb). Axis 2 is not shown in the figure; it separated samples by sampling time (spring vs. autumn). Ctrl, control; CPMS, composted sludge; FS, fiber sludge; LPMS, lime‐stabilized sludge

The soil amendments increased basal respiration in spring and microbial biomass (estimated by the fumigation‐extraction technique) in autumn (Table 2). Clear changes in bacterial and fungal communities (Figure 3; Supplemental Tables S9 and S10), revealed by DNA‐based amplicon sequencing were observed in soils that received the amendments, although the coarser phospholipid fatty acid method indicated that microbial biomass remained rather unchanged (Supplemental Table S11). The treatments also affected the amount of K_2_SO_4_–extractable C in soil, but the changes interacted with the season (Supplemental Table S11). The levels of K_2_SO_4_–extractable N and glomalin‐related soil proteins were only marginally affected by the treatments (Table 2).

**TABLE 2:** Basal soil respiration (BR), microbial biomass C (C_MB_) and N (N_MB_), and glomalin‐related soil proteins (GRSP) in control and treatment plots (*n* = 5)

Treatment^a^	BR	C_MB_	N_MB_	GRSP^b^
	μg CO_2_ kg^−1^ h^−1^	mg kg^−1^		g kg^−1^
Spring samples				
FS	95.8 (82.7–111)***	0.18 (0.16–0.21)	0.027 (0.022–0.032)	
LPMS	61.9 (53.4–71.7)	0.18 (0.16–0.20)	0.028 (0.024–0.033)	
CPMS	91.0 (78.6–106)**	0.17 (0.15–0.19)	0.027 (0.022–0.031)	
Control	56.6 (48.9–65.6)	0.16 (0.15–0.18)	0.028 (0.023–0.032)	
Autumn samples				
FS	42.0 (36.3–48.7)	0.25 (0.22–0.28)**	0.046 (0.041–0.051)**	1.71 (1.43–1.99)^†^
LPMS	42.6 (36.8–49.4)	0.25 (0.23–0.28)**	0.046 (0.042–0.051)**	1.55 (1.27–1.83)
CPMS	45.6 (39.3–52.8)	0.25 (0.22–0.28)**	0.042 (0.037–0.047)**	1.63 (1.35–1.91)
Control	45.4 (39.2–52.7)	0.20 (0.18–0.22)	0.032 (0.027–0.037)	1.42 (1.14–1.70)

*Note*. Values in parentheses are 95% confidence intervals. Asterisks indicate significant difference between treatment and control.

^a^ CPMS, composted sludge; FS, fiber sludge; LPMS, lime‐stabilized sludge.

^b^ Determined for autumn samples but not for spring samples.

^*^Significant at the .05 probability level.

^**^Significant at the .01 probability level.

^***^Significant at the .001 probability level.

^†^Significant at the .1 pobability level.

Sampling time and amendment type explained roughly 8 and 19% of the variation in microbial community composition (*p *< .001) of both bacterial and fungal operational taxonomic units, respectively (Figure 3; Supplemental Table S12). All amendments increased soil pH (Supplemental Table S4), which seemed to be an important environmental factor affecting the bacterial and fungal communities in the soil (Figure 3). The bacterial and fungal communities in the LPMS treatment, with the highest soil pH, differed most from the control and had the highest microbial N and microbial C (Figure 3; Table 2; Supplemental Table S4). Closer analysis of the microbial communities (Supplemental Table S9) revealed a distinct increase in plant root‐associated fungal species, such as Sebacinales (around 300–700% increase in log‐scale) and the arbuscular mycorrhizal fungus *Funneliformis mossae*, in plots receiving soil amendments. Operational taxonomic units representing various bacterial groups showed significant differences between the treatments and control plots (Supplemental Table S10). Bacterial representatives clustering into eight taxa *(Anaerolineae*, *Chitinophagaceae*, *Demequinaceae*, *Microscillaceae*, *Myxococcales*, *Pedosphaeraceae*, *Rhodanobacteraceae*, and *Xanthomonadaceae*) were more common in all amended plots, showing 200–700% increases (in log‐scale, *p* Adj. ≤ .0001) compared with the unamended control.

## 4 DISCUSSION

Rainfall simulation was conducted with an intensity of 5 mm h^−1^, which represents typical rains in Finland (Kuusisto, 1980). Although heavier storms naturally cause severe erosion events if occurring when soils are bare, the persistent but less spectacular losses of clay particles mobilized by lower intensity rains maintain turbidity of rivers flowing through the landscapes of southwestern Finland. This relatively flat area has predominantly clay‐textured, structured soils and requires pipe drains to carry the weight of machines in spring. Thus, after frost disappears, most of the excess water discharges as subsurface drainage flow (Koskiaho et al., 2002; Turtola & Paajanen, 1995). Our rainfall simulation setup aimed to mimic the generation of subsurface drainage discharge during typical rainy periods.

Amending soil with pulp mill side streams decreased particle mobilization to percolation water and associated P losses over multiple years. As compared to other soil amendments used as erosion or PP control agents, our results suggest that the pulp mill wastes bring about as large reductions in SS and PP as gypsum or highly reactive lime [CaO/Ca(OH)_2_, so called “structural lime”] applications have done in earlier Finnish and Swedish studies (Ekholm et al., 2012; Svanbäck, Ulén, & Etana, 2014; Ulén & Etana, 2014). Ekholm et al. (2012) reported 64% reduction in PP exports from a 245‐ha catchment having 100 ha of agricultural land that was almost all amended with gypsum at 4 Mg ha^−1^ and monitored after that for 3 yr. Ulén and Etana (2014) reported a 40–57% reduction in TP losses from two field sites after application of reactive lime at a rate of 5 Mg ha^−1^ CaO equivalents. In the 6‐yr study of Svanbäck et al. (2014), reactive lime applied to annually plowed soil decreased PP losses by one‐third.

Because soil amendments used in our study had a large C/P ratio, mobilization of DRP was not observed but DRP concentration in percolation water were in all treatments practically the same as that of percolates from control soil. Ekholm et al. (2012) estimated that gypsum amendment would have decrease DRP loss by about 30% during their study, because marked elevation of EC of soil solution suppresses P desorption from soil particle surfaces. For reactive lime, Ulén and Etana (2014) reported 10–40% reduction in DRP loss from the two field sites. Svanbäck et al. (2014) measured equal annual DRP losses for control plots and those treated with reactive lime.

The mechanism behind the observed multi‐year improvement in surface soil stability is likely more complicated with the addition of fiber sludge materials than in the case of inorganic, soluble soil‐improving materials. In a previous study conducted using the same experimental set‐up with intact soil monoliths cored for indoor rainfall simulations from gypsum‐amended fields, Uusitalo et al. (2012) found that gypsum significantly elevated EC in the soil solution and in percolation water (>300 μS cm^−1^), which promoted aggregate stability and flocculation of clay particles. The effect on SS concentration was dependent on how long it took for gypsum to leach out of the soil profile. In the present study, addition of sludges from the pulp and paper industry also increased EC (Supplemental Figure S2) but not to the same degree as gypsum. We hypothesize that, in the case of organic soil amendments used in the present study, direct interactions of soil minerals with the added particulate organic matter and microbe‐derived compounds stabilizing aggregates played a major role (see discussion below). The effect was also at least as long‐lived as the effect brought about by gypsum application (Ekholm et al., 2012; Uusitalo et al., 2012).

Due to their high C/nutrient ratios, these soil amendments can be used in much higher quantities than many organic materials that are more nutrient rich, such as manures and composted biosolids. However, attempts to increase soil C content may lead to increased leaching of C, especially on soils with higher C stocks. The present results indicate an initial flush of DOC that subsides with time. Declining DOC in percolation water may indicate fast microbial decomposition of the organic matter added to soil and/or stabilization of particulate organic matter within soil aggregates, thus immobilizing them in soil.

Only a small proportion of the added C was recovered in the soil at the end of the 4‐yr study (Supplemental Table S4), leaning to rapid microbial turnover of the added organic matter. The most easily degradable organic matter is broken down during composting, whereas more recalcitrant compounds end up in the soil (Heikkinen et al., unpublished data, 2020; Hubbe, Nazhad, & Sánchez, 2010). In line with those findings, a small increase in soil C concentration was only measured for the CPMS treatment. In boreal mineral soils, the mean annual C decline is estimated to be 0.4% relative to the existing C concentration (data from Finnish cropland soils for the period 1974–2009; Heikkinen et al., 2013). If the amendments tested in the present study had decomposed at this rate, almost all of the C applied would have been recovered after 4 yr. The almost 8 Mg of nonrecovered applied C indicated that losses of added C were two orders of magnitude greater than average decline of 0.4% in soil organic matter in Finnish soils.

In our study, relative proportions of numerous microbial groups were changed compared with the control treatment 3 yr after addition of pulp and paper industry side streams. It is shown that changes even at the species level can alter the chemical composition of extracellular polymeric substances produced by microorganisms (Costa et al., 2018) and that different microbial species have different effects on soil aggregation and erosion (Lehmann et al., 2020; Rigardo & Troeh 1979). In the present study, observed changes in microbial community were well in line with our hypothesis. However, to what extend microbiological interactions with soil minerals could explain improved soil stability against water‐induced stresses remains open.

Although the concept of aggregate stabilization due to microbial excretion was established almost a century ago (Peele & Beale, 1941), recent studies have revealed new details relating to stabilization of soil particles and C sequestration. Kopittke et al. (2018, 2020) showed that microbe‐derived, N‐rich organic compounds in particular are capable of forming new organo‐mineral associations on the surface of mineral particles. In the present study, microbial‐bound N increased in all treated soils (Table 2).

Lavallee, Soong, and Cotrufo (2020) addressed the importance of conceptualization of soil organic matter pools in order to deepen understanding of organic matter functioning, persistence, and formation. In their approach, mineral‐associated organic matter is distinguished from free particulate organic matter, and mineral‐associated organic matter fraction is considered to be physically protected against microbial turnover. Recently, Lehmann et al. (2020) reported that the fungal strains showing a dense growth of mycelia possessed the highest probability of aggregate formation, and most of these effective aggregator strains belonged to the phylum Ascomycota. Apart from Sebacinaceae, almost all of the most abundantly increased fungi in our study belonged to Ascomycota. For example, *Tetracladium marchalianum*, which showed a 230% increase (in log‐scale) under all treatments (Supplemental Table S9), was one of the most efficient aggregator fungi according to Lehmann et al. (2020). In addition, microbial necromass was recently estimated to make up more than half of soil organic matter in temperate soils (Liang et al., 2019). These new findings suggest that the effects of added organic matter on soil properties are largely mediated by soil microbiology. Thus, the reduction in particle mobilization observed in the present study might be associated with activation of soil microbiota and a subsequent increase in the proportion of mineral‐associated organic matter, which stabilizes soil aggregates. However, because the data only reveal positive associations rather than deeper mechanistic features, this remains speculation. More detailed studies are required to confirm whether the quality and stability of soil C is affected by organic soil amendments.

The processing of the organic material (lignocellulosic material processed in elevated temperature) appeared to directly affect fungal species found in soil, as thermotolerant species (e.g. *Thermomyces lanuginosus*; Singh, Madlala, & Prior, 2003) and species decomposing lignin and cellulose (e.g., *Mycothermus thermophilus*; Natvig, Taylor, Tsang, Hutchinson, & Powell, 2015) were abundant in LPMS and CPMS treatments. Among other abundant fungi, Sebacinales are suggested to indicate the less intensive land use typical of organic farming (Verbruggen et al., 2014), and *F. mossae* is found to improve nutrient status and biomass of ryegrass (Berthelo, Blaudez, Beguiristain, Chalot, & Leyval, 2018). Also, many bacteria groups that are beneficial for agricultural soils were detected, such as parasites on other bacteria (Bdellovibrionaceae; Starr & Baigent, 1966) and aerobic chemoheterotrophs mineralizing organic C from plant biomass (Chthoniobacteraceae; Kant et al., 2011). They were also abundant in the soils that received pulp and paper industry side streams (Supplemental Table S10). It is tempting to speculate that their rise could be connected to qualitative changes in soil caused by the amendments.

Overall, the studied organic side streams had only a minor effect on yields, and Cd present in amendments did not accumulate in soil or grains. In the study of Price and Voroney (2007), significant changes in soil heavy metal concentration after multiple applications of papermill biosolids were not found either. However, caution should be exercised if repeated applications are planned. In practice, Cd concentrations in side streams from the pulp and paper industry vary between individual mills, which allows selection of soil amendment materials with low Cd content for use in agricultural applications.

## 5 CONCLUSIONS

This 4‐yr field‐scale experiment indicated that FS and composted and lime‐stabilized sludge from the pulp and paper industry can be used to mitigate adverse effects of food production to the quality of discharge waters. The amendments showed a potential to reduce soil erosion through soil monoliths over several years. They also increased the pools of microbial‐bound C and N in soil. A particularly interesting observation was an increased proportion of Sebacinales fungi, which are used as indicators of improved quality of agricultural soil in organic farming. However, the organic soil amendments tested had only minor effects on cereal yield and grain quality. The only concern with their use related to the Cd contents of the amendments. Although soil and grain Cd content was not affected in this study, care must be taken to select appropriate sources of materials with low Cd content. Further research on the contributions of industrial and forest‐derived organic side streams to soil microbiological functions and their relations to food production and long‐term C sequestration are required.

## AUTHOR CONTRIBUTIONS

Kimmo Rasa: Conceptualization; Data curation; Formal analysis; Funding acquisition; Investigation; Methodology; Project administration; Writing‐original draft; Writing‐review & editing. Taina Pennanen: Conceptualization; Data curation; Formal analysis; Investigation; Methodology; Writing‐original draft; Writing‐review & editing. Krista Peltoniemi: Conceptualization; Data curation; Formal analysis; Investigation; Methodology; Writing‐original draft; Writing‐review & editing. Sannakajsa Velmala: Conceptualization; Data curation; Formal analysis; Investigation; Methodology; Writing‐original draft; Writing‐review & editing. Hannu Fritze: Conceptualization; Data curation; Formal analysis; Funding acquisition; Investigation; Methodology; Visualization; Writing‐original draft; Writing‐review & editing. Janne Kaseva: Data curation; Formal analysis; Methodology; Software; Writing‐original draft; Writing‐review & editing. Juuso Joona: Funding acquisition; Resources; Writing‐original draft; Writing‐review & editing. Risto Uusitalo: Conceptualization; Data curation; Formal analysis; Funding acquisition; Investigation; Methodology; Project administration; Visualization; Writing‐original draft; Writing‐review & editing.

## CONFLICT OF INTEREST

The authors declare no conflict of interest.

## Supporting information

SUPPORTING MATERIAL
