# Supplementary material for: Pulp and paper mill sludges decrease soil erodibility
Source: J Environ Qual. 2020 Dec 8;50(1):172–84. doi: 10.1002/jeq2.20170 (PMC7898543; doi:10.1002/jeq2.20170)
Supplement: Supplementary file 1 — SUPPORTING MATERIAL [file JEQ2-50-172-s001.docx]

**Supplementary Material to:**

**Pulp and Paper Mill Sludges Decrease Soil Erodibility**

Kimmo Rasa^a*^, Taina Pennanen^b^, Krista Peltoniemi^b^, Sannakajsa Velmala^b^, Hannu Fritze^b^, Janne Kaseva^a^, Juuso Joona^c^, and Risto Uusitalo^a^

^a^ Natural Resources Institute Finland, Tietotie 4, FI-31600 Jokioinen, Finland

^b^ Natural Resources Institute Finland, Latokartanonkaari 9, FI-00790 Helsinki, Finland

^c^ Soilfood Oy, Viikinkaari 6, FI-00790 Helsinki, Finland

*Corresponding author ([kimmo.rasa@luke.fi](mailto:kimmo.rasa@luke.fi))

**Pulp and paper industry side streams used in the experiment, their origin, properties, and application rates**

The materials used in the experiment were obtained from Stora Enso Imatra Mills by Tyynelän maanparannus Oy (currently Soilfood Oy) in autumn 2015. These materials were 1) composted pulp mill sludge (CPMS), 2) lime-stabilized pulp mill sludge (LPMS), both derived from the biological water treatment process of the factory, and 3) fiber sludge (FS) originating from pre-clarifying of cardboard process waters. Pulp mill sludge (PMS) was taken from the water treatment system of the plant in June 2015. The CPMS was composted in a static pile for two months, while the LPMS was hygienized by adding a 5% mixture of slaked and burned lime and then stored in a pile for two months. Under Finnish legislation, composting or lime stabilization is required for hygiene reasons before application of PMS to agricultural soils. FS is a hygienic material and was derived directly from the Imatra Mill without any pretreatment. The main difference between the materials was that LPMS and CPMS contained phosphorus, nitrogen and other elements from the wood and pulp mill processes, while FS is a nutrient-poor cellulose-based organic material (Table S1).

To determine water-soluble nitrogen (N) and phosphorus (P), composite samples in their original moisture content were mixed with water (1:5 v/v, 1 h shaking), filtered through paper filters (Tesorb 80 g m^2^; Tervakoski, Finland), and analyzed using a continuous flow analyzer (Skalar San++ System). Total N was determined in a subsample of the same extract after Kjeldahl digestion with H_2_SO_4_, CuSO_4_, and K_2_SO_4_. Dewarda’s alloy was used in analysis of total N. Dried materials were analyzed for their main nutrient and heavy metal content (SFS-EN 13650 aqua regia extraction, ICP-OES Perkin Elmer Optima 8300), ash content (SFS 3008, loss of ignition at 550 °C), carbon (C) and N content (dry combustion, Leco CN2000). Material pH and electrical conductivity (EC) were detected from 1:5 solid to water slurry (SFS-EN 13037, SFS-EN 13038).

The amendment rate of fresh materials was 52090, 51370 and 72155 kg ha^-1^ for LPMS, CPMS, and FS, respectively. The aim of amendment was to increase soil carbon content taking into account current regulations with respect to use of soil amendments. Thus, the restriction on the amendment rates was national legislation, which limits application of soluble N to 30 kg ha^-1^ in autumn (Table 1). Due to the minor amount of nutrients in FS (soluble N not a limiting factor), the application rate was fixed to approximately 8 tons of organic carbon. Application rates were estimated on the basis of preliminary data and the actual inputs based on material analysis are presented in Table 1. Use of these amendments passed the legislative cadmium (Cd) content threshold of 1.5 mg kg^-1^ (FS 0.01, LPMS 0.60 and CPMS 0.96 mg Cd kg^-1^ dm). However, the high application rate of CPMS and LPMS resulted in the application rate of Cd exceeding the maximum permissible cumulative Cd level for a five-year period (7.5 g Cd ha^-1^). In practice, the chemical composition of paper mill sludge varies between factories, which shows the importance of quality control when products are applied to agricultural fields. By selecting sludges with lower Cd content, high amendment rates could be used in practice.

Table S1. Selected properties of the organic side streams from the pulp and paper industry used as soil amendments (CPMS = composted sludge, LPMS = lime-stabilized sludge, FS = fiber sludge). Dry matter (DM) and carbon content (C), total N content (Tot N), content of water-extractable (1:5 W V^-1^) nitrogen (Sol N) and phosphate-phosphorus (Sol PO_4_), and content of aqua regia-extractable nutrients and heavy metals.

|  | Units | CPMS | LPMS | FS |
| --- | --- | --- | --- | --- |
| DM | % | 43.4 | 49.7 | 33.5 |
| C | % | 35.1 | 34.8 | 34.9 |
| Ash | % | 35.9 | 33.2 | 34.8 |
| EC | µS cm^-1^ | 584 | 874 | 98.8 |
| pH |  | 7.9 | 8.0 | 8.7 |
| Tot N | g kg^-1^ | 9.5 | 9.8 | 0.5 |
| Sol N | g kg^-1^ | 1.6 | 1.3 | 0.0 |
| Sol PO_4_ | mg kg^-1^ | 23.7 | 25.1 | 0.1 |
| Al | g kg^-1^ | 16.8 | 13.9 | 2.7 |
| As | mg kg-1 | 1.1 | 1.9 | 0.3 |
| Ca | g kg^-1^ | 43 | 84 | 94 |
| Cd | mg kg^-1^ | 0.96 | 0.60 | 0.01 |
| Co | mg kg^-1^ | 1.6 | 1.0 | 0.3 |
| Cr | mg kg^-1^ | 33 | 20 | 4 |
| Cu | mg kg^-1^ | 24 | 18 | 2 |
| Fe | g kg^-1^ | 4.1 | 4.0 | 0.2 |
| Hg | mg kg^-1^ | 0.05 | 0.06 | 0.02 |
| K | g kg^-1^ | 1.73 | 1.17 | 0.03 |
| Mg | g kg^-1^ | 1.8 | 2.1 | 0.8 |
| Mn | mg kg^-1^ | 1504 | 1141 | 11 |
| Mo | mg kg^-1^ | 1.41 | 1.58 | 0.42 |
| Ni | mg kg^-1^ | 10.7 | 5.6 | 0.7 |
| P | g kg^-1^ | 2.01 | 2.04 | 0.07 |
| Pb | mg kg^-1^ | 7.8 | 6.3 | 0.8 |
| S | g kg^-1^ | 4.9 | 5.1 | 0.3 |
| Zn | mg kg^-1^ | 215 | 116 | 2 |

**Field experiment: establishment fertilization, plant protection and management**

The field was established in September 2015. Materials were weighed and spread manually by hand to secure even application. Thereafter, the soil was cultivated to about 10 cm depth (Kongskilde 2250). In subsequent years, similar cultivation was conducted after harvesting. In each spring, seedbed preparation was carried out using a harrow (depth approx. 3-5 cm), after which the field was fertilized (Table S2) and sown. In 2016 the crop was wheat (Anniina), in 2017 oats (Obelix), in 2018 oats (Venla), and in 2019 wheat (Anniina).

Nitrogen fertilization rates used in the experiment were within the local recommended ranges for N fertilization. In autumn 2015, about 30 kg soluble N were added to the soil in the CPMS and LPMS treatments. This was compensated for in spring 2016 by adding 30 kg more N in the FS and Ctrl treatments. According to earlier studies on soil initial P reserves suggest that the yield potential of the field is not sensitive to P fertilization rate (Valkama et al. 2009). Although P fertilization was not expected to affect yield potential in the experimental soil, P fertilizer was applied in the FS and Ctrl treatments (CPMS and LPMS contain P). The P dosage given, along with high amendment rate of CPMS and LPMS, was considered reserve fertilization covering a five-year period.

Table S2. Annual fertilizer application rates (kg ha^-1^) in control (Ctrl) and amended plots (CPMS = composted sludge, LPMS = lime-stabilized sludge, FS = fiber sludge) in the field experiment, and plant protection chemicals used in the experiment

|  | 2016 | ___2017___ | | ___2018___ | | ___2019___ | |
| --- | --- | --- | --- | --- | --- | --- | --- |
|  | N | N | P | N | P | N | P |
| CPMS | 90 | 80 | - | 85 | - | 85 | - |
| LPMS | 90 | 80 | - | 85 | - | 85 | - |
| FS | 120 | 85 | 10 | 90 | 10 | 90 | 10 |
| Ctrl | 120 | 85 | 10 | 90 | 10 | 90 | 10 |
| Plant  protection | Express,  Starane | Ariane | | Ally 50 ST,  Primus | | Premium Classic, Starane | |

**Rainfall simulation**

*Determination of macropore volume of undisturbed soil monoliths*

The clay soils of SW Finland are sediments of the different stages of the Baltic Sea, with horizontal strata of soil matter of coarser (material settled during spring flow) and finer (material settled in calm water in winter) texture. These strata are the natural planes of horizontal cleavage and they are relatively stable against dispersion, unlike a cut surface.

After coring the monoliths, they were inverted for preparation of the bottom, and if the bottom had a cut surface (they were most often broken according to the natural cleavage planes), the natural cleavage plane was exposed by carefully inserting a blunt knife some millimeters in the soil and levered until soil broke to a smooth surface. An intact surface or ped faces was then exposed and the bottom vacuumed to remove crumbs and any loose material. The space between the lower edge of the sampling cylinder and the actual soil was measured in 2 lines (12 points) across the area of the cylinder and void space filled with quartz sand. The bottom was secured with a fine nylon netting and turned to upright position. In a similar way, the distance of the upper edge of the cylinder and soil was measured. This way we know the volume of the soil in a cored monolith.

The monolith was thereafter placed to a frame/chassis/bed with a drainage hose. When saturating the soils, the level of the drainage hose was adjusted using a rail to the level of the soil monolith bottom. By measuring the mass of water that drains overnight after a soil has been saturated to the surface level, we can calculate the volume of large pores that conduct water. They are in Supplemental Figure S1 taken as >30 µm pores.

**Microbiological analyses**

*Basal respiration, microbial biomass C and N, and K_2_SO_4_-extractable C and N*

The soil samples were kept at 14°C for two days prior to the respiration measurements. The basal respiration rate was determined on fresh soil (20 mL) as the amount of CO_2_ evolved after 24 h incubation, measured as described by Pietikäinen & Fritze (1995). The amount of C and N in the microbial biomass in soil samples was determined by chloroform fumigation-extraction as described in Törmänen et al. (2018). Carbon and N flushes from the microbial biomass were determined as the difference between the fumigated and unfumigated samples and converted to microbial biomass C (C_MB_) and microbial biomass N (N_MB_) as mg of C or N per kg of dry soil. Non-fumigated K_2_SO_4_-extractable C and N correspond to labile forms of soil C (C_EXT_) and N (N_EXT_).

*DNA extraction, amplicon sequencing, PLFA and bioinformatics*

PipeCraft utilizes several implemented tools, e.g., mothur v1.36.1 (Schloss et al. 2009), vsearch v1.11.1, CD-HIT v4.6 (Fu et al. 2012) and swarm v2.1.8 (Mahé et al. 2015), in pre-processing, assembling, chimera filtering, and clustering steps. Raw sequence reads were processed according to the manual with slight modifications for demultiplexed sequence data. In brief, assembly of paired end reads and initial quality filtering was conducted with vsearch (v1.11.1; github.com/torognes/vsearch; Rognes et al. 2016) according to the following parameters: minimum overlap 15, max differences 99, minimum length 150bp, e_max 1, max ambiguous 0 and trunc qual 10 and 20 for bacteria and fungi, respectively. On average 38% of the raw reads were filtered out in the assembly. After renaming the reads and creating group files and merging sequence and group files, sequences were reoriented to 5’-3’direction. Chimera filtering was performed for the reoriented reads by using vsearch (v1.11.1) de novo filtering with parameters: annotation 0.97 and abskew 2; and for ITS reference-based filtering was used with Unite ITS2 ref v7.1 as the database. Primers and primer artefacts were filtered out from sequences at this step. In addition, fungal ITS2 region was extracted from reads with ITSx (Bengtsson-Palme et al. 2013). This resulted in 1197208 bacterial reads that were affiliated to 9272 operational taxonomic units (OUT) and 1108545 fungal reads that were affiliated to 2887 OTUs.

In the next step, sequence reads were clustered and an OTU table was created with CD-hit with parameters: threshold 0.97 and min size 2. In the final step, bacterial OTUs were taxonomically annotated by searching representative sequences with BLAST using reference 16S rRNA (SILVA_123_SSURef_Nr99_tax_silva.fasta) obtained from SILVA (Quast et al. 2013; Yilmaz et al. 2014). For fungi, the ITS2 database (sh_genral_release_dynamic_01.12.2018.fasta) from UNITE (Nilsson et al. 2018) was used.

Bacterial and fungal OTUs that had high e-value (>e-25) and low query coverage (<90%) and identity with the bacterial (<90%) and fungal (<70%) database match were filtered out. OTUs that had affiliation other than bacteria or fungi, singleton OTUs, and reads with relative proportion below 0.0001% from the total sum of reads were also removed from the data. Bacterial OTUs with the same GenBank accession numbers and fungal OTUs referring to the exact same species hypothesis (Kõljalg et al. 2013) were consolidated. The final filtering resulted in 1131828 bacterial reads that affiliated to 3334 OTUs and 871303 fungal reads that affiliated to 803 OTUs. Raw sequence data have been deposited in the NCBI genebank sequence archive (SRA) BioProject PRJNA607883, with accession numbers SAMN14150014-53.

Phospholipid fatty acid (PLFA) analysis was carried out as described by Frostegård et al. (1993) using 2.5 g of fresh soil, and analyzed and named according to Pennanen et al. 1999. In total, 43 different PLFAs were identified from each sample and used to calculate the microbial biomass indicators PLFA_total_, PLFA_bact_, and PLFA_fung_ (Pennanen et al. 1999).

**Supplemental results**


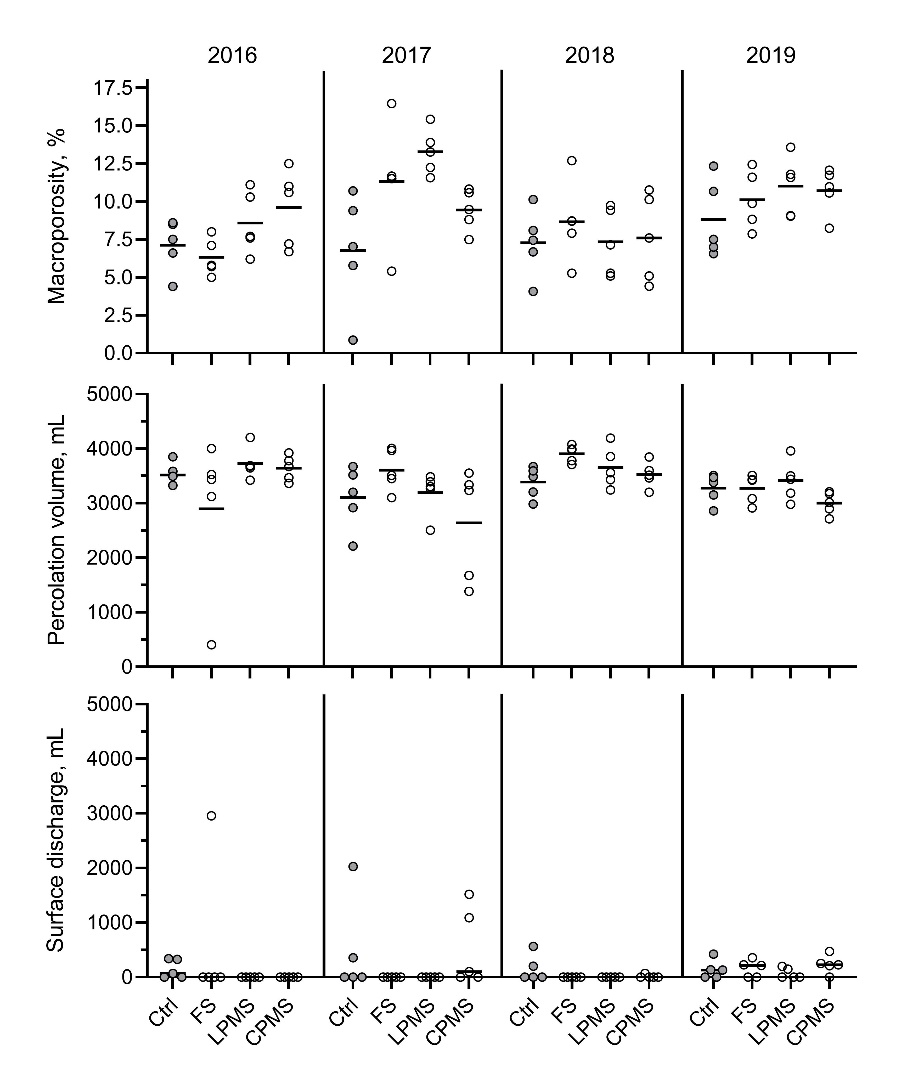


Supplemental Figure S1. Macroporosity (pores > 30 µm in diameter, see method above), total volume of percolation water and volume of runoff water in rainfall simulation test (error bars denote measured minimum and maximum values).

Table S3**.** Pairwise comparisons of differences between control (Ctrl) and treatments (LPMS = lime-stabilized sludge, CPMS = composted sludge, FS = fiber sludge) over the four-year study period in rainfall simulation data (percolation water chemistry). Values shown are difference in Least Square Means, with adjusted *p*-values and 95% confidence intervals. For parameter abbreviations, see main text

|  | |  | ____________PP____________ | | | | | ____________TP____________ | | | |
| --- | --- | --- | --- | --- | --- | --- | --- | --- | --- | --- | --- |
| Treatments | | | Est. | p | | Conf. interval | | Est. | p | Conf. interval |  |
| FS | LPMS | | 0.03 | 0.98 | | -0.19 | 0.25 | -0.02 | 0.99 | -0.24 | 0.20 |
| FS | Ctrl | | -0.76 | <.0001 | | -0.97 | -0.54 | -0.66 | <.0001 | -0.88 | -0.43 |
| FS | CPMS | | -0.30 | 0.0033 | | -0.52 | -0.08 | -0.23 | 0.04 | -0.46 | -0.01 |
| LPMS | Ctrl | | -0.79 | <.0001 | | -1.01 | -0.57 | -0.64 | <.0001 | -0.86 | -0.41 |
| LPMS | CPMS | | -0.33 | 0.0001 | | -0.55 | -0.11 | -0.21 | 0.07 | -0.43 | 0.01 |
| Ctrl | CPMS | | 0.45 | <.0001 | | 0.24 | 0.67 | 0.43 | <.0001 | 0.20 | 0.65 |
|  |  | | ____________DRP__________ | | | | | ____________SS____________ | | | |
| Treatments | | | Est. | p | Conf. interval | | | Est. | p | Conf. interval | |
| FS | LPMS | | -0.18 | 0.91 | -0.92 | | 0.57 | 13 | 0.94 | -50 | 77 |
| FS | Ctrl | | -0.15 | 0.94 | -0.90 | | 0.60 | -243 | <.0001 | -307 | -180 |
| FS | CPMS | | 0.07 | 0.99 | -0.68 | | 0.82 | -84 | 0.01 | -148 | -20 |
| LPMS | Ctrl | | 0.02 | 1.00 | -0.72 | | 0.77 | -257 | <.0001 | -321 | -193 |
| LPMS | CPMS | | 0.25 | 0.78 | -0.50 | | 0.99 | -98 | 0.0016 | -161 | -34 |
| Ctrl | CPMS | | 0.22 | 0.83 | -0.53 | | 0.97 | 159 | <.0001 | 95 | 223 |
|  |  | | ____________TN__________ | | | | | __________ NO_3_-N __________ | | | |
| Treatments | | | Est. | p | Conf. interval | | | Est. | p | Conf. interval | |
| FS | LPMS | | -1.20 | 0.02 | -2.26 | | -0.13 | -0.84 | 0.06 | -1.71 | 0.04 |
| FS | Ctrl | | -0.51 | 0.54 | -1.57 | | 0.55 | -0.06 | 1.00 | -0.94 | 0.81 |
| FS | CPMS | | -0.36 | 0.77 | -1.42 | | 0.70 | -0.12 | 0.98 | -0.99 | 0.75 |
| LPMS | Ctrl | | 0.68 | 0.27 | -0.34 | | 1.71 | 0.77 | 0.08 | -0.08 | 1.63 |
| LPMS | CPMS | | 0.83 | 0.13 | -0.19 | | 1.86 | 0.71 | 0.12 | -0.14 | 1.57 |
| Ctrl | CPMS | | 0.15 | 0.98 | -0.87 | | 1.17 | -0.06 | 1.00 | -0.91 | 0.80 |
|  |  | | ___________ NH_4_-N ________ | | | | | _________Turbidity________ | | | |
| Treatments | | | Est. | p | Conf. interval | | | Est. | p | Conf. interval | |
| FS | LPMS | | 0.01 | 1.00 | -0.36 | | 0.38 | 53 | 0.73 | -84 | 189 |
| FS | Ctrl | | -0.12 | 0.81 | -0.49 | | 0.25 | -368 | <.0001 | -504 | -231 |
| FS | CPMS | | 0.00 | 1.00 | -0.37 | | 0.37 | -112 | 0.14 | -249 | 25 |
| LPMS | Ctrl | | -0.13 | 0.77 | -0.50 | | 0.24 | -420 | <.0001 | -557 | -284 |
| LPMS | CPMS | | -0.01 | 1.00 | -0.37 | | 0.36 | -165 | 0.01 | -302 | -28 |
| Ctrl | CPMS | | 0.12 | 0.79 | -0.25 | | 0.49 | 255 | <.0001 | 119 | 392 |
|  |  | | _________ ___TC_____ ____ | | | | | ____________DOC____________ | | | |
| Treatments | | | Est. | p | Conf. interval | | | Est. | p | Conf. interval | |
| FS | LPMS | | -0.08 | 1.00 | -8.34 | | 8.19 | 0.46 | 0.99 | -4.33 | 5.25 |
| FS | Ctrl | | 15.96 | 0.0001 | 7.69 | | 24.23 | 5.56 | 0.02 | 0.77 | 10.35 |
| FS | CPMS | | 8.81 | 0.03 | 0.54 | | 17.08 | 2.79 | 0.37 | -2.00 | 7.58 |
| LPMS | Ctrl | | 16.03 | 0.0001 | 7.77 | | 24.30 | 5.10 | 0.03 | 0.31 | 9.89 |
| LPMS | CPMS | | 8.88 | 0.03 | 0.62 | | 17.15 | 2.34 | 0.52 | -2.45 | 7.12 |
| Ctrl | CPMS | | -7.15 | 0.11 | -15.42 | | 1.12 | -2.77 | 0.38 | -7.56 | 2.02 |
|  |  | | ____________IC____________ | | | |  | ____________S____________ | | | |
| Treatments | | | Est. | p | | Conf. interval | | Est. | p | Conf. interval | |
| FS | LPMS | | -0.53 | 0.98 | | -4.59 | 3.52 | -2.05 | <.0001 | -2.64 | -1.46 |
| FS | Ctrl | | 10.39 | <.0001 | | 6.34 | 14.45 | -0.06 | 0.99 | -0.65 | 0.53 |
| FS | CPMS | | 6.01 | 0.0031 | | 1.96 | 10.07 | -1.01 | 0.0009 | -1.60 | -0.42 |
| LPMS | Ctrl | | 10.93 | <.0001 | | 6.87 | 14.98 | 1.99 | <.0001 | 1.39 | 2.58 |
| LPMS | CPMS | | 6.55 | 0.0014 | | 2.49 | 10.60 | 1.04 | 0.0007 | 0.44 | 1.63 |
| Ctrl | CPMS | | -4.38 | 0.03 | | -8.44 | -0.33 | -0.95 | 0.0016 | -1.54 | -0.36 |
|  |  | | ____________EC____________ | | | | | ____________pH____________ | | | |
| Treatments | | | Est. | p | Conf. interval | | | pH | p | Conf. interval | |
| FS | LPMS | | -11.58 | 0.91 | -63.44 | | 40.28 | -0.03 | 0.85 | -0.13 | 0.07 |
| FS | Ctrl | | 93.16 | 0.0008 | 41.30 | | 145.0 | 0.23 | <.0001 | 0.13 | 0.33 |
| FS | CPMS | | 46.07 | 0.09 | -5.80 | | 97.93 | 0.12 | 0.01 | 0.02 | 0.22 |
| LPMS | Ctrl | | 104.74 | 0.0003 | 52.88 | | 156.6 | 0.26 | <.0001 | 0.16 | 0.36 |
| LPMS | CPMS | | 57.65 | 0.03 | 5.78 | | 109.5 | 0.15 | 0.0019 | 0.05 | 0.25 |
| Ctrl | CPMS | | -47.09 | 0.08 | -98.96 | | 4.77 | -0.11 | 0.03 | -0.21 | -0.01 |
|  |  | | ____________Ca____________ | | | | | ____________K____________ | | | |
| Treatments | | | Est. | p | Conf. interval | | | Est. | p | Conf. interval | |
| FS | LPMS | | -2.36 | 0.39 | -6.63 | | 1.91 | -0.20 | 0.10 | -0.44 | 0.03 |
| FS | Ctrl | | 6.15 | 0.01 | 1.88 | | 10.42 | 0.20 | 0.12 | -0.03 | 0.43 |
| FS | CPMS | | 2.50 | 0.35 | -1.77 | | 6.77 | -0.05 | 0.93 | -0.28 | 0.18 |
| LPMS | Ctrl | | 8.51 | 0.0004 | 4.25 | | 12.78 | 0.40 | 0.0002 | 0.17 | 0.63 |
| LPMS | CPMS | | 4.86 | 0.02 | 0.59 | | 9.13 | 0.15 | 0.31 | -0.08 | 0.38 |
| Ctrl | CPMS | | -3.65 | 0.10 | -7.92 | | 0.62 | -0.25 | 0.03 | -0.48 | -0.02 |
|  |  | | ____________Mg____________ | | | | |  |  |  |  |
| Treatments | | | Est. | p | Conf. interval | | |  |  |  |  |
| FS | LPMS | | -0.22 | 0.99 | -3.40 | | 2.96 |  |  |  |  |
| FS | Ctrl | | 1.93 | 0.06 | -0.14 | | 4.00 |  |  |  |  |
| FS | CPMS | | 1.29 | 0.15 | -0.64 | | 3.22 |  |  |  |  |
| LPMS | Ctrl | | 2.15 | 0.10 | -0.61 | | 4.91 |  |  |  |  |
| LPMS | CPMS | | 1.51 | 0.21 | -1.14 | | 4.17 |  |  |  |  |
| Ctrl | CPMS | | -0.64 | 0.20 | -1.74 | | 0.47 |  |  |  |  |

Table S4. Carbon (C, %), nitrogen (N, %), cadmium (Cd) concentration, electrical conductivity (EC), and pH of control (Ctrl) and treatment soils (LPMS = lime-stabilized sludge, CPMS = composted sludge, FS = fiber sludge) at the end of the experiment (depth of 0-20 cm, n=5). CL Low and CL Upp refer to lower and upper 95% confidence interval, P values refer to difference between treatment and control.

|  | __________C__________ | | | | __________N__________ | | | | _________Cd__________ | | | |
| --- | --- | --- | --- | --- | --- | --- | --- | --- | --- | --- | --- | --- |
| Treat-ment | % | CL Low | CL  Upp | p | % | CL Low | CL  Upp | p | mg kg^-1^ | CL Low | CL  Upp | p |
| FS | 2.34 | 2.20 | 2.47 | 0.767 | 0.17 | 0.16 | 0.18 | 0.679 | 0.16 | 0.15 | 0.17 | 0.984 |
| LPMS | 2.40 | 2.27 | 2.54 | 0.388 | 0.18 | 0.17 | 0.19 | 0.679 | 0.16 | 0.16 | 0.17 | 0.611 |
| CPMS | 2.50 | 2.36 | 2.63 | 0.053 | 0.18 | 0.17 | 0.19 | 0.480 | 0.17 | 0.16 | 0.17 | 0.558 |
| Ctrl | 2.32 | 2.18 | 2.45 |  | 0.18 | 0.17 | 0.19 |  | 0.16 | 0.15 | 0.17 |  |
|  | _________EC__________ | | | | _________pH__________ | | | |  |  |  |  |
| Treat-ment | mS cm^-1^ | CL Low | CL  Upp | p | pH | CL Low | CL  Upp | p |  |  |  |  |
| FS | 0.87 | 0.83 | 0.91 | <.0001 | 6.81 | 6.70 | 6.92 | <.0001 |  |  |  |  |
| LPMS | 0.83 | 0.79 | 0.87 | <.0001 | 6.69 | 6.59 | 6.80 | <.0001 |  |  |  |  |
| CPMS | 0.71 | 0.66 | 0.75 | 0.001 | 6.40 | 6.30 | 6.51 | 0.005 |  |  |  |  |
| CTRL | 0.61 | 0.56 | 0.65 |  | 6.25 | 6.14 | 6.36 |  |  |  |  |  |


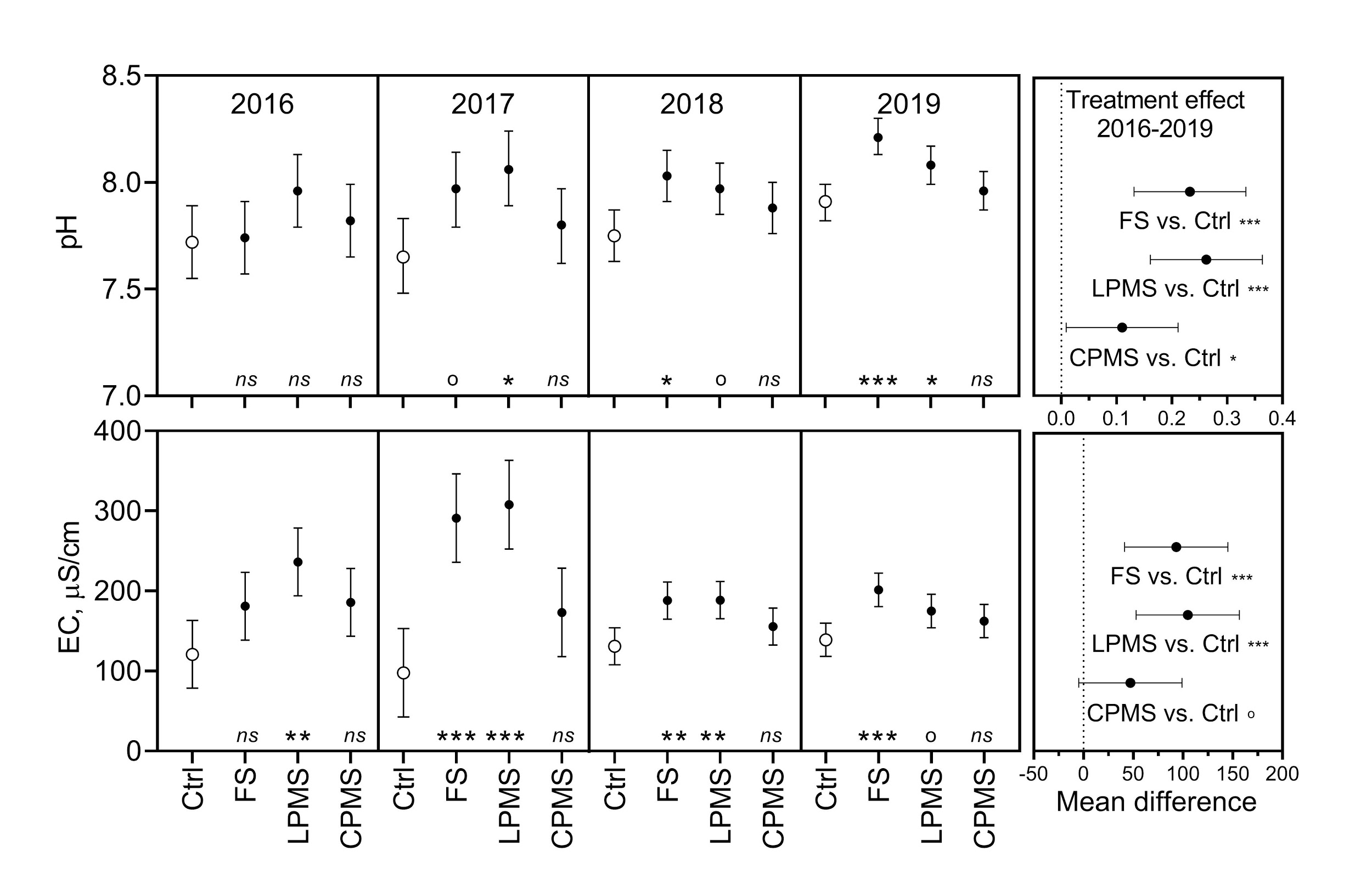


Figure S2. pH and electrical conductivity (EC) in percolation water leached from 40-cm deep monoliths of control (Ctrl) and treated soil (LPMS = lime-stabilized sludge, CPMS = composted sludge, FS = fiber sludge). Treatment effect over the 4-year experimental period is summarized in the right-hand panel (error bars denote confidence limits). ***p<0.001, **p<0.01, *p<0.05, ^O^p<0.1, ns p>0.1.

Table S5. Concentrations of ammonium-nitrogen (NH4-N), total carbon (TC) and inorganic C (IC), and turbidity in percolation water leached from 40-cm deep monoliths of control (Ctrl) and treated soil (LPMS = lime-stabilized sludge, CPMS = composted sludge, FS = fiber sludge). CL = 95% confidence interval, and P values refer to differences between treatment and control.

|  |  | _____________NH_4_-N___________ | | | | _____________TC_____________ | | | |
| --- | --- | --- | --- | --- | --- | --- | --- | --- | --- |
| Treatment | Year | mg L^-1^ | CL lower | CL Upper | p | mg L^-1^ | CL lower | CL Upper | p |
| FS | 2016 | 0.08 | 0.05 | 0.13 | 0.9535 | 49.8 | 35.0 | 64.6 | 0.1065 |
| FS | 2017 | 0.10 | 0.07 | 0.13 | 0.9281 | 41.7 | 34.3 | 49.1 | 0.0036 |
| FS | 2018 | 0.17 | 0.14 | 0.20 | 0.9242 | 34.1 | 28.4 | 39.8 | 0.3498 |
| FS | 2019 | 0.09 | 0.08 | 0.11 | 0.9281 | 32.7 | 28.1 | 37.2 | 0.1853 |
| LPMS | 2016 | 0.09 | 0.06 | 0.16 | 0.9535 | 48.7 | 33.9 | 63.5 | 0.1208 |
| LPMS | 2017 | 0.10 | 0.07 | 0.12 | 0.9077 | 51.7 | 44.3 | 59.1 | <.0001 |
| LPMS | 2018 | 0.17 | 0.14 | 0.20 | 0.9084 | 31.2 | 25.5 | 36.8 | 0.7085 |
| LPMS | 2019 | 0.08 | 0.06 | 0.10 | 0.3376 | 27.0 | 22.5 | 31.5 | 0.9354 |
| CPMS | 2016 | 0.07 | 0.04 | 0.12 | 0.9535 | 39.0 | 24.2 | 53.9 | 0.4006 |
| CPMS | 2017 | 0.10 | 0.08 | 0.13 | 0.9297 | 30.5 | 23.1 | 37.9 | 0.2062 |
| CPMS | 2018 | 0.17 | 0.14 | 0.21 | 0.9297 | 27.4 | 21.8 | 33.1 | 0.98 |
| CPMS | 2019 | 0.09 | 0.08 | 0.11 | 0.9297 | 26.0 | 21.5 | 30.6 | 0.98 |
| Ctrl | 2016 | 0.08 | 0.05 | 0.14 |  | 22.4 | 7.6 | 37.2 |  |
| Ctrl | 2017 | 0.12 | 0.09 | 0.15 |  | 19.4 | 12.0 | 26.8 |  |
| Ctrl | 2018 | 0.19 | 0.16 | 0.24 |  | 27.1 | 21.5 | 32.8 |  |
| Ctrl | 2019 | 0.10 | 0.09 | 0.13 |  | 25.5 | 20.9 | 30.0 |  |
|  |  | _____________IC_____________ | | | | __________Turbidity__________ | | | |
| Treatment | Year | mg L^-1^ | CL lower | CL Upper | p | NTU | CL lower | CL Upper | p |
| FS | 2016 | 18.7 | 14.2 | 23.1 | 0.0662 | 389 | 187 | 592 | 0.0052 |
| FS | 2017 | 25.1 | 19.6 | 30.5 | 0.0006 | 292 | 68 | 516 | 0.0007 |
| FS | 2018 | 18.7 | 15.7 | 21.6 | 0.0156 | 261 | 174 | 348 | 0.0258 |
| FS | 2019 | 17.9 | 15.4 | 20.4 | 0.0003 | 201 | 91 | 310 | 0.0177 |
| LPMS | 2016 | 21.5 | 17.1 | 26.0 | 0.0111 | 191 | -11 | 394 | <.0001 |
| LPMS | 2017 | 30.8 | 25.3 | 36.2 | <.0001 | 211 | -13 | 435 | 0.0001 |
| LPMS | 2018 | 16.7 | 13.7 | 19.7 | 0.1109 | 198 | 110 | 285 | 0.0007 |
| LPMS | 2019 | 13.4 | 10.9 | 15.9 | 0.1128 | 332 | 223 | 442 | 0.2871 |
| CPMS | 2016 | 16.9 | 12.5 | 21.4 | 0.1301 | 374 | 171 | 576 | 0.0041 |
| CPMS | 2017 | 13.7 | 8.2 | 19.1 | 0.1301 | 648 | 424 | 871 | 0.1623 |
| CPMS | 2018 | 14.0 | 11.1 | 17.0 | 0.4903 | 282 | 195 | 369 | 0.0685 |
| CPMS | 2019 | 11.6 | 9.1 | 14.1 | 0.4903 | 288 | 179 | 398 | 0.1623 |
| Ctrl | 2016 | 10.5 | 6.0 | 14.9 |  | 876 | 673 | 1078 |  |
| Ctrl | 2017 | 5.8 | 0.4 | 11.3 |  | 940 | 716 | 1164 |  |
| Ctrl | 2018 | 12.3 | 9.3 | 15.2 |  | 395 | 308 | 482 |  |
| Ctrl | 2019 | 10.2 | 7.7 | 12.7 |  | 403 | 294 | 513 |  |

Table S6. Concentrations of calcium (Ca), potassium (K), magnesium (Mg), and sulfur (S) in percolation water leached from 40-cm deep monoliths of control (Ctrl) and treated soil (LPMS = lime-stabilized sludge, CPMS = composted sludge, FS = fiber sludge). CL = 95% confidence interval, P values refer to difference between treatment and control.

|  |  | _____________Ca_____________ | | | | _____________K______________ | | | |
| --- | --- | --- | --- | --- | --- | --- | --- | --- | --- |
| Treatment | Year | mg l^-1^ | CL lower | CL Upper | p | mg l-1 | CL lower | CL Upper | p |
| FS | 2016 | 21.5 | 16.9 | 26.2 | 0.0094 | 2.0 | 1.5 | 2.6 | 0.3949 |
| FS | 2018 | 21.4 | 16.8 | 26.1 | 0.0193 | 1.8 | 1.4 | 2.4 | 0.3949 |
| FS | 2019 | 17.6 | 13.0 | 22.3 | 0.8752 | 1.8 | 1.3 | 2.4 | 0.7918 |
| LPMS | 2016 | 29.2 | 24.6 | 33.9 | <.0001 | 3.1 | 2.3 | 4.1 | 0.0003 |
| LPMS | 2018 | 20.3 | 15.7 | 25.0 | 0.0449 | 1.9 | 1.4 | 2.5 | 0.2206 |
| LPMS | 2019 | 18.1 | 13.5 | 22.8 | 0.8752 | 2.0 | 1.5 | 2.6 | 0.5964 |
| CPMS | 2016 | 22.7 | 18.0 | 27.3 | 0.0032 | 2.5 | 1.9 | 3.4 | 0.0131 |
| CPMS | 2018 | 15.5 | 10.8 | 20.1 | 0.7928 | 1.6 | 1.2 | 2.2 | 0.617 |
| CPMS | 2019 | 14.9 | 10.3 | 19.6 | 0.8057 | 1.8 | 1.4 | 2.4 | 0.7918 |
| Ctrl | 2016 | 12.2 | 7.6 | 16.9 |  | 1.5 | 1.2 | 2.0 |  |
| Ctrl | 2018 | 13.0 | 8.3 | 17.6 |  | 1.4 | 1.1 | 1.9 |  |
| Ctrl | 2019 | 16.9 | 12.3 | 21.6 |  | 1.6 | 1.2 | 2.2 |  |
|  |  | _____________Mg_____________ | | | | _____________S______________ | | | |
| Treatment | Year | mg l-1 | CL lower | CL Upper | p | mg l-1 | CL lower | CL Upper | p |
| FS | 2016 | 8.19 | 6.15 | 0K | 0.1264 | 2.2 | 1.6 | 2.9 | 0.9939 |
| FS | 2018 | 7.90 | 5.87 | 0K | 0.2239 | 2.8 | 2.1 | 3.4 | 0.9939 |
| FS | 2019 | 8.59 | 6.55 | 0K | 0.4861 | 4.7 | 4.0 | 5.3 | 0.9939 |
| LPMS | 2016 | 9.32 | 7.59 | Cast | 0.0068 | 7.3 | 6.6 | 8.0 | <.0001 |
| LPMS | 2018 | 8.09 | 6.36 | Cast | 0.1008 | 4.4 | 3.7 | 5.0 | 0.0092 |
| LPMS | 2019 | 7.93 | 6.19 | Cast | 0.4861 | 4.1 | 3.5 | 4.8 | 0.6465 |
| CPMS | 2016 | 7.50 | 6.58 | Kom | 0.0303 | 4.6 | 4.0 | 5.3 | <.0001 |
| CPMS | 2018 | 6.66 | 5.74 | Kom | 0.4443 | 3.9 | 3.2 | 4.5 | 0.1344 |
| CPMS | 2019 | 6.65 | 5.73 | Kom | 0.4861 | 4.2 | 3.6 | 4.9 | 0.6929 |
| Ctrl | 2016 | 5.73 | 4.81 | Ctrl |  | 2.3 | 1.7 | 3.0 |  |
| Ctrl | 2018 | 5.85 | 4.94 | Ctrl |  | 2.8 | 2.2 | 3.5 |  |
| Ctrl | 2019 | 7.31 | 6.40 | Ctrl |  | 4.7 | 4.1 | 5.4 |  |

**
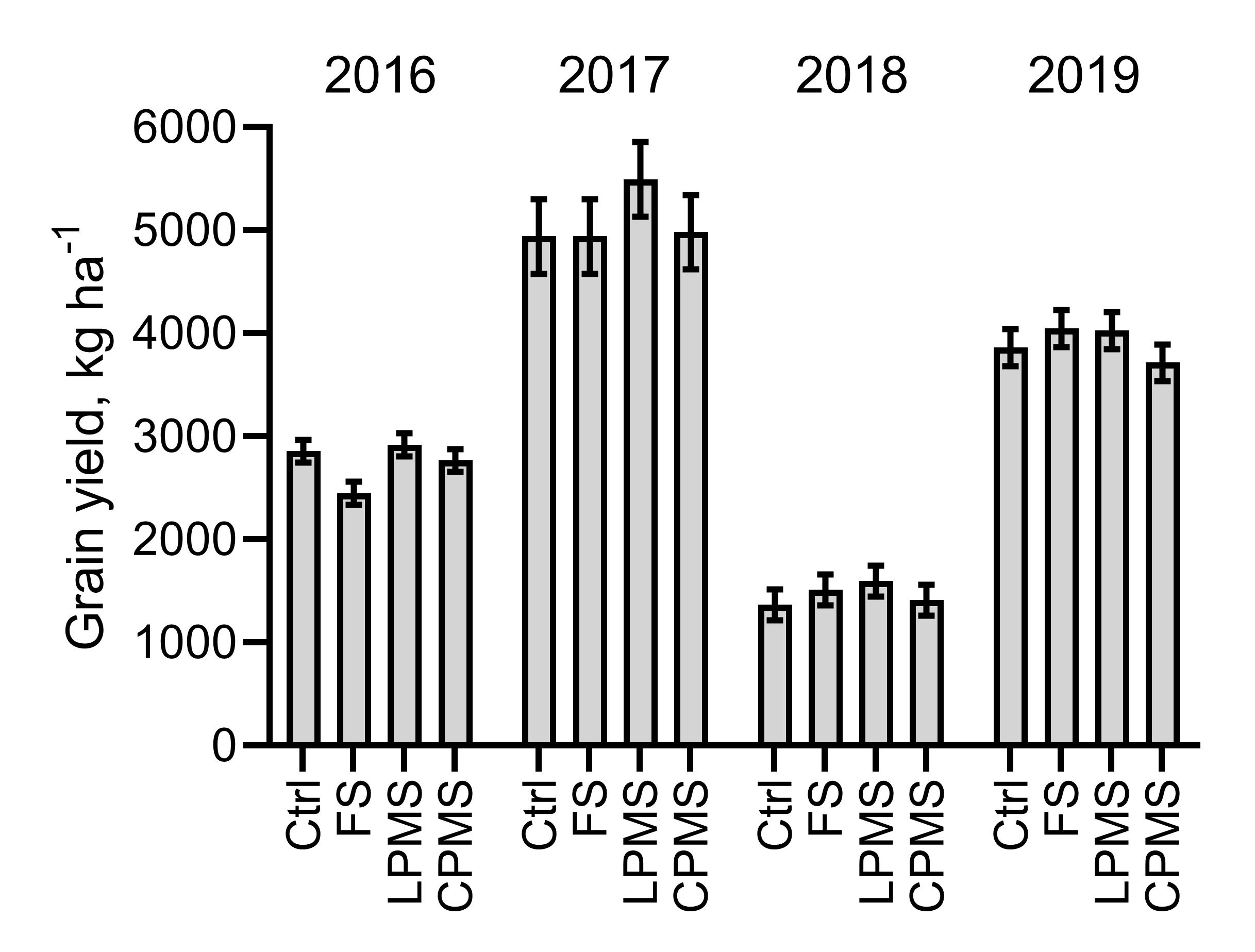
**

Figure S3. Annual grain yield expressed as dry matter weight. Error bars denote the 95% confidence interval. No statistically significant differences in annual yield between treatments (LPMS = lime-stabilized sludge, CPMS = composted sludge, FS = fiber sludge) and the control (Ctrl) were observed (p>0.1), except between Ctrl and FS in 2016 (p<0.0001).

Table S7. Concentration of nutrients and heavy metals in grain in 2016 and 2017 (mean and standard deviation) in the control (Ctrl) and treatment plots (LPMS = lime-stabilized sludge, CPMS = composted sludge, FS = fiber sludge). < = below detection limit, na. = not available.

|  |  | _____2016 mean values_____ | | | | _____2017 mean values_____ | | | |
| --- | --- | --- | --- | --- | --- | --- | --- | --- | --- |
|  |  | FS | LPMS | CPMS | Ctrl | FS | LPMS | CPMS | Ctrl |
| Ca | g kg^-1^ | 0.32 | 0.34 | 0.30 | 0.33 | 0.47 | 0.49 | 0.46 | 0.44 |
| Cu | mg kg^-1^ | 6.0 | 5.9 | 5.9 | 6.2 | 2.8 | 2.7 | 2.7 | 2.7 |
| Fe | mg kg^-1^ | 45 | 46 | 44 | 47 | 66 | 66 | 64 | 65 |
| K | g kg^-1^ | 6.4 | 6.4 | 6.3 | 6.3 | 4.9 | 4.8 | 4.8 | 4.8 |
| Mg | g kg^-1^ | 1.9 | 1.9 | 1.8 | 1.9 | 1.3 | 1.3 | 1.3 | 1.3 |
| Mn | mg kg^-1^ | 42.4 | 34.0 | 42.2 | 50.8 | 24.0 | 26.2 | 31.4 | 36.4 |
| P | g kg^-1^ | 5.4 | 5.6 | 5.3 | 5.5 | 3.8 | 3.8 | 3.8 | 3.7 |
| S | g kg^-1^ | 1.7 | 1.9 | 1.8 | 1.8 | 1.3 | 1.4 | 1.3 | 1.3 |
| Se | µg kg^-1^ | 16.2 | 11.7 | 11.1 | 15.7 | na. | na. | na. | na. |
| Zn | mg kg^-1^ | 43.6 | 46.8 | 46.8 | 48.0 | 22.6 | 21.4 | 21.8 | 22.2 |
| Al | mg kg^-1^ | 6.5 | 0.8 | 1.0 | 1.1 | na. | na. | na. | na. |
| As | µg kg^-1^ | <26 | <26 | <26 | <26 | <24 | <24 | <24 | <24 |
| Cd | µg kg^-1^ | 47.4 | 58.8 | 38.2 | 43.8 | <13 | <13 | <13 | <13 |
| Co | µg kg^-1^ | <26 | <26 | <26 | <26 | <24 | <24 | <24 | <24 |
| Cr | mg kg^-1^ | 0.31 | 0.31 | 0.33 | 0.33 | 0.09 | 0.10 | 0.09 | 0.09 |
| Mo | µg kg^-1^ | 1320 | 450 | 538 | 880 | 1518 | 1019 | 828 | 836 |
| Ni | µg kg^-1^ | 230 | 214 | 220 | 242 | 978 | 918 | 1016 | 1420 |
| Pb | µg kg^-1^ | <13 | <13 | <13 | <13 | 73.6 | 75.2 | 71.6 | 60.8 |
|  |  | ________2017 stdev________ | | | | ________2017 stdev________ | | | |
|  |  | FS | LPMS | CPMS | Ctrl | FS | LPMS | CPMS | Ctrl |
| Ca | g kg^-1^ | 0.02 | 0.00 | 0.03 | 0.03 | 0.01 | 0.03 | 0.02 | 0.02 |
| Cu | mg kg^-1^ | 0.19 | 0.34 | 0.15 | 0.25 | 0.08 | 0.13 | 0.08 | 0.13 |
| Fe | mg kg^-1^ | 1.9 | 1.2 | 3.4 | 3.4 | 3.2 | 4.4 | 2.5 | 3.6 |
| K | g kg^-1^ | 0.29 | 0.26 | 0.23 | 0.24 | 0.21 | 0.21 | 0.15 | 0.10 |
| Mg | g kg^-1^ | 0.10 | 0.08 | 0.07 | 0.08 | 0.02 | 0.05 | 0.05 | 0.02 |
| Mn | mg kg^-1^ | 3.8 | 4.4 | 1.9 | 0.8 | 2.1 | 3.3 | 0.9 | 1.8 |
| P | g kg^-1^ | 0.33 | 0.21 | 0.27 | 0.31 | 0.09 | 0.19 | 0.18 | 0.17 |
| S | g kg^-1^ | 0.12 | 0.03 | 0.11 | 0.13 | 0.06 | 0.04 | 0.05 | 0.06 |
| Se | µg kg^-1^ | 3.3 | 3.8 | 4.0 | 5.3 | na. | na. | na. | na. |
| Zn | mg kg^-1^ | 3.1 | 3.1 | 2.5 | 3.2 | 1.3 | 1.7 | 1.1 | 1.1 |
| Al | mg kg^-1^ | 12.0 | 0.4 | 0.1 | 0.1 | na. | na. | na. | na. |
| As | µg kg^-1^ | - | - | - | - | - | - | - | - |
| Cd | µg kg^-1^ | 22.1 | 39.8 | 6.9 | 2.3 | - | - | - | - |
| Co | µg kg^-1^ | - | - | - | - | - | - | - | - |
| Cr | mg kg^-1^ | 0.02 | 0.03 | 0.01 | 0.02 | 0.02 | 0.01 | 0.01 | 0.01 |
| Mo | µg kg^-1^ | 327 | 68 | 92 | 135 | 304 | 188 | 76 | 175 |
| Ni | µg kg^-1^ | 37 | 61 | 23 | 37 | 129 | 270 | 142 | 363 |
| Pb | µg kg^-1^ | - | - | - | - | 15.4 | 14.0 | 15.5 | 14.0 |

Table S8. Concentration of nutrients and heavy metals in grain in 2018 and 2019 (mean and standard deviation) in the control (Ctrl) and treatment plots (LPMS = lime-stabilized sludge, CPMS = composted sludge, FS = fiber sludge). < = below detection limit, na. = not available.

|  |  | _____2018 mean values_____ | | | | _____2019 mean values_____ | | | |
| --- | --- | --- | --- | --- | --- | --- | --- | --- | --- |
|  |  | FS | LPMS | CPMS | Ctrl | FS | LPMS | CPMS | Ctrl |
| Ca | g kg^-1^ | 0.71 | 0.68 | 0.69 | 0.68 | 0.31 | 0.30 | 0.30 | 0.29 |
| Cu | mg kg^-1^ | 4.3 | 4.3 | 4.5 | 4.4 | 4.9 | 4.9 | 5.1 | 4.9 |
| Fe | mg kg^-1^ | 161 | 155 | 168 | 169 | 50 | 50 | 49 | 50 |
| K | g kg^-1^ | 5.8 | 5.5 | 5.7 | 5.6 | 4.2 | 4.2 | 4.2 | 4.2 |
| Mg | g kg^-1^ | na. | na. | na. | na. | 1.5 | 1.5 | 1.5 | 1.5 |
| Mn | mg kg^-1^ | 27.2 | 28.3 | 34.8 | 38.4 | 17.7 | 18.3 | 24.2 | 30.8 |
| P | g kg^-1^ | 5.1 | 5.0 | 4.9 | 4.9 | 4.2 | 4.1 | 4.1 | 4.1 |
| S | g kg^-1^ | 2.1 | 2.2 | 2.1 | 2.1 | 1.6 | 1.6 | 1.6 | 1.5 |
| Se | µg kg^-1^ | na. | na. | na. | na. | 16.5 | 16.2 | 14.8 | 13.3 |
| Zn | mg kg^-1^ | 37.1 | 37.7 | 40.6 | 39.5 | 31.3 | 33.9 | 36.3 | 35.8 |
| Al | mg kg^-1^ | 8.0 | 3.9 | 7.2 | 8.7 | 5.1 | 3.2 | 2.8 | 4.9 |
| As | µg kg^-1^ | 52.2 | 44.3 | 49.0 | 44.7 | <139 | <139 | <139 | <139 |
| Cd | µg kg^-1^ | 13.9 | 12.2 | 13.5 | 12.8 | 43.6 | 36.8 | 34.3 | 35.7 |
| Co | µg kg^-1^ | <23 | <23 | <23 | <23 | <15 | <15 | <15 | <15 |
| Cr | mg kg^-1^ | 0.21 | 0.17 | 0.17 | 0.18 | 0.27 | 0.31 | 0.46 | 0.27 |
| Mo | µg kg^-1^ | 867 | 594 | 357 | 340 | 1176 | 811 | 552 | 462 |
| Ni | µg kg^-1^ | 3 | 2 | 3 | 3 | 178 | 200 | 186 | 286 |
| Pb | µg kg^-1^ | 55.6 | 56.7 | 65.4 | 52.7 | <112 | <112 | <112 | <112 |
|  |  | ________2018 stdev________ | | | | ________2019 stdev________ | | | |
|  |  | FS | LPMS | CPMS | CTRL | FS | LPMS | CPMS | CTRL |
| Ca | g kg^-1^ | 0.03 | 0.01 | 0.02 | 0.02 | 0.01 | 0.01 | 0.01 | 0.01 |
| Cu | mg kg^-1^ | 0.05 | 0.28 | 0.10 | 0.19 | 0.16 | 0.13 | 0.31 | 0.18 |
| Fe | mg kg^-1^ | 16.8 | 6.3 | 5.0 | 16.3 | 2.7 | 2.5 | 2.4 | 1.7 |
| K | g kg^-1^ | 0.24 | 0.11 | 0.09 | 0.24 | 0.09 | 0.04 | 0.15 | 0.16 |
| Mg | g kg^-1^ | na. | na. | na. | na. | 0.03 | 0.02 | 0.07 | 0.05 |
| Mn | mg kg^-1^ | 1.3 | 1.6 | 1.7 | 1.6 | 2.2 | 2.0 | 2.5 | 2.1 |
| P | g kg^-1^ | 0.15 | 0.16 | 0.15 | 0.14 | 0.06 | 0.03 | 0.15 | 0.11 |
| S | g kg^-1^ | 0.06 | 0.06 | 0.04 | 0.04 | 0.03 | 0.04 | 0.08 | 0.03 |
| Se | µg kg^-1^ | na. | na. | na. | na. | 4.4 | 6.1 | 3.1 | 2.8 |
| Zn | mg kg^-1^ | 1.3 | 2.3 | 1.1 | 0.7 | 1.6 | 1.4 | 3.4 | 1.2 |
| Al | mg kg^-1^ | 9.9 | 2.0 | 5.0 | 7.5 | 2.3 | 2.6 | 3.2 | 3.2 |
| As | µg kg^-1^ | 12.5 | 9.8 | 6.2 | 8.7 | - | - | - | - |
| Cd | µg kg^-1^ | 1.5 | 0.9 | 2.1 | 0.6 | 3.3 | 3.0 | 5.1 | 2.9 |
| Co | µg kg^-1^ | - | - | - | - | - | - | - | - |
| Cr | mg kg^-1^ | 0.09 | 0.02 | 0.02 | 0.03 | 0.02 | 0.08 | 0.19 | 0.07 |
| Mo | µg kg^-1^ | 138 | 130 | 61 | 90 | 115 | 120 | 56 | 106 |
| Ni | µg kg^-1^ | 0.2 | 0.4 | 0.3 | 0.4 | 41 | 75 | 40 | 52 |
| Pb | µg kg^-1^ | 6.1 | 5.8 | 12.5 | 4.8 | - | - | - | - |

Table S9. Fungal indicator OTUs at species level in differential abundance analysis (DESeq2) showing significant >1.7 fold change (p adj <0.001 or <0.05). Results are presented as paired comparisons, positive values indicate higher abundance in control (Ctrl) and negative values higher abundance in treatment plots (LPMS = lime-stabilized sludge, CPMS = composted sludge, FS = fiber sludge).

| Ctrl vs. FS | log_2_ fold change | p adj |
| --- | --- | --- |
| Sebacinaceae_sp | -6.91149 | 3.42E-12 |
| Dactylella_arnaudii | -5.90384 | 2.58E-05 |
| Paecilomyces_penicillatus | -5.19369 | 6.20E-07 |
| Tetracladium_sp.2 | -5.09617 | 0.000556 |
| Sebacinales_sp | -4.79188 | 6.20E-07 |
| Arthrobotrys_vermicola | -4.65611 | 0.000601 |
| Serendipitaceae_sp | -3.77281 | 5.74E-13 |
| Savoryella_appendiculata | -2.51873 | 3.67E-08 |
| Occultifur_externus | -2.51397 | 1.33E-06 |
| Tetracladium_marchalianum | -2.19342 | 1.62E-14 |
| Cercozoa_sp.1 | -2.18056 | 0.000341 |
| Operculomyces_laminatus | -2.08139 | 2.47E-05 |
| Funneliformis_mosseae | -1.75856 | 0.021188 |
| Kochiomyces_sp | 1.713487 | 1.75E-05 |
| Ascomycota_sp.5 | 1.945431 | 0.000556 |
| Basidiomycota_sp.2 | 2.374667 | 0.000631 |
| Sordariales_sp.3 | 2.864353 | 3.71E-06 |
| Sordariales_sp.2 | 3.561875 | 3.67E-08 |
| Orbiliaceae_sp.2 | 4.091277 | 0.000341 |
| Agaricales_sp | 6.165184 | 1.46E-12 |
| Ctrl vs. LPMS | log_2_ fold change | p adj |
| Mycothermus_thermophilus | -9.48029 | 7.30E-40 |
| Coprinopsis_sp | -8.84751 | 3.12E-24 |
| Chaetomiaceae_sp | -8.54734 | 1.66E-27 |
| Stachybotrys_chartarum | -8.1753 | 5.74E-20 |
| Thermomyces_lanuginosus | -8.026 | 8.40E-26 |
| Sebacinales_sp | -6.46604 | 3.81E-13 |
| Fungi_sp.5 | -5.14309 | 2.73E-05 |
| Sordariales_sp.7 | -5.09625 | 1.66E-05 |
| Aspergillaceae_sp | -4.6955 | 8.16E-05 |
| Psilocybe_inquilina | -4.22548 | 0.000916 |
| Sebacinaceae_sp | -3.2174 | 0.015448 |
| Penicillium_solitum | -2.89081 | 0.000185 |
| Savoryella_appendiculata | -2.48525 | 1.16E-07 |
| Tetracladium_marchalianum | -2.33547 | 2.11E-16 |
| Funneliformis_mosseae | -2.03616 | 0.003984 |
| Operculomyces_laminatus | -1.98129 | 0.000126 |
| Kochiomyces_sp | 1.721174 | 2.79E-05 |
| Ascomycota_sp.5 | 1.994537 | 0.000605 |
| Sordariales_sp.3 | 3.467861 | 2.53E-07 |
| Sordariales_sp.2 | 3.892091 | 1.03E-08 |
| Mastigobasidium_sp | 4.126922 | 0.00081 |
| Orbiliaceae_sp.2 | 4.278817 | 0.000289 |
| Ctrl vs. CPMS | log_2_ fold change | p adj |
| Fungi_sp.5 | -7.14386 | 8.35E-11 |
| Stachybotrys_chartarum | -6.81701 | 7.93E-14 |
| Thermomyces_lanuginosus | -5.27471 | 8.35E-11 |
| Sebacinales_sp | -5.1524 | 3.64E-08 |
| Apiotrichum_dulcitum | -4.63709 | 1.23E-22 |
| Coprinopsis_sp | -4.63033 | 3.31E-06 |
| Aspergillaceae_sp | -4.4573 | 0.000201 |
| Mycothermus_thermophilus | -4.16252 | 1.32E-06 |
| Sebacinaceae_sp | -3.45921 | 0.004892 |
| Funneliformis_mosseae.1 | -2.38584 | 0.039271 |
| Thelebolus_globosus | -2.31937 | 9.10E-06 |
| Tetracladium_marchalianum | -2.30594 | 1.43E-16 |
| Ascobolus_sp | -2.28892 | 3.64E-08 |
| Serendipitaceae_sp | -1.99924 | 0.000912 |
| Savoryella_appendiculata | -1.97105 | 3.92E-05 |
| Funneliformis_mosseae | -1.65637 | 0.027528 |
| Sordariales_sp.3 | 1.643901 | 0.011801 |
| Sordariales_sp.2 | 1.979881 | 0.002901 |

Table S10. Bacterial indicator OTUs at species level of order or family (if affiliation could be obtained from the database) in differential abundance analysis (DESeq2) showing significant (>2 fold change, p adj <0.001) groups. Results are presented as paired comparisons, positive values indicate higher abundance in control (CTRL) and negative values higher abundance in treatment plots (LPMS = lime-stabilized sludge, CPMS = composted sludge, FS = fiber sludge).

| Ctrl vs. FS | log_2_ fold change | p adj |
| --- | --- | --- |
| Micropepsaceae | 3.9 | 0.001 |
| Xanthomonadaceae | 3.2 | 0.0002 |
| Myxococcales | 2.3 | 2.50E-08 |
| Acidobacteriaceae Subgroup 1 | 2.2 | 0.001 |
| Nitrosomonadaceae | -2.0 | 9.70E-08 |
| Flavobacteriales | -2.1 | 0.0002 |
| Solibacteraceae Subgroup 3 | -2.1 | 0.00003 |
| Pirellulaceae | -2.2 | 0.0002 |
| Myxococcaceae | -2.2 | 0.00008 |
| Anaerolineae | -2.2 | 9.50E-07 |
| Rubritaleaceae | -2.2 | 1.60E-06 |
| Blastocatellaceae | -2.3 | 0.0004 |
| Beijerinckiaceae | -2.3 | 0.0004 |
| Pirellulaceae | -2.3 | 0.0003 |
| Microscillaceae | -2.4 | 2.80E-21 |
| Azospirillaceae | -2.4 | 1.00E-10 |
| Microscillaceae | -2.5 | 3.40E-15 |
| Pedosphaeraceae | -2.6 | 1.60E-14 |
| Microscillaceae | -2.6 | 0.00001 |
| Microscillaceae | -2.7 | 0.00001 |
| Chthoniobacteraceae | -2.7 | 4.10E-07 |
| Demequinaceae | -2.7 | 3.80E-18 |
| Pirellulaceae | -2.8 | 0.0002 |
| Anaerolineae | -2.8 | 0.0002 |
| Sandaracinaceae | -2.9 | 0.0002 |
| Phycisphaeraceae | -2.9 | 0.0001 |
| Anaerolineae | -3.0 | 0.00001 |
| Pseudohongiellaceae | -3.1 | 3.20E-15 |
| Myxococcales | -3.3 | 1.30E-15 |
| Microscillaceae | -3.3 | 2.00E-12 |
| Rhodanobacteraceae | -3.4 | 0.0002 |
| Betaproteobacteriales | -3.6 | 6.60E-09 |
| Bdellovibrionaceae | -3.6 | 0.00002 |
| Chitinophagaceae | -3.6 | 2.00E-16 |
| Myxococcales | -3.6 | 1.00E-11 |
| Planctomycetes | -3.6 | 0.0004 |
| Xanthomonadaceae | -3.7 | 1.20E-12 |
| Microscillaceae | -3.7 | 0.00006 |
| Anaerolineae | -3.7 | 0.00004 |
| Microscillaceae | -3.7 | 4.00E-08 |
| Myxococcales | -3 | 0.001 |
| Hyphomonadaceae | -3.7 | 0.00008 |
| Sandaracinaceae | -3.7 | 0.00002 |
| Rhodobacteraceae | -3.8 | 0.0002 |
| Chitinophagaceae | -3.9 | 0.00004 |
| Anaerolineae | -3.9 | 4.70E-09 |
| Nannocystaceae | -3.9 | 0.0002 |
| Micromonosporaceae | -4.0 | 0.00004 |
| Nannocystaceae | -4.0 | 0.00002 |
| Gemmatimonadaceae | -4.0 | 2.70E-06 |
| Haliangiaceae | -4.0 | 1.20E-06 |
| Microscillaceae | -4.1 | 0.00005 |
| Microscillaceae | -4.1 | 3.10E-15 |
| Phycisphaeraceae | -4.2 | 0.0003 |
| Longimicrobiaceae | -4.2 | 0.00002 |
| Rhodanobacteraceae | -4.3 | 3.70E-07 |
| Rhodanobacteraceae | -4.4 | 4.70E-06 |
| Gammaproteobacteria | -4.5 | 3.90E-07 |
| Rhodanobacteraceae | -4.6 | 4.70E-07 |
| Pedosphaeraceae | -4.7 | 2.70E-08 |
| Verrucomicrobiaceae | -4.8 | 2.00E-08 |
| Rhodanobacteraceae | -4.9 | 3.20E-09 |
| Xanthomonadaceae | -5.1 | 6.00E-14 |
| Chitinophagaceae | -5.1 | 1.50E-10 |
| Anaerolineae | -5.1 | 3.70E-10 |
| Rhodanobacteraceae | -5.2 | 3.30E-10 |
| Myxococcales | -5.9 | 1.50E-12 |
| Myxococcales | -6.4 | 2.40E-16 |
| Ctrl vs. LPMS | log_2_ fold change | p adj |
| Xanthomonadaceae | 4.3 | 6.20E-06 |
| Solibacteraceae Subgroup 3 | 4.1 | 0.0005 |
| Pirellulaceae | 2.6 | 0.0001 |
| Sphingobacteriaceae | 2.2 | 0.001 |
| Isosphaeraceae | 2.2 | 0.0004 |
| Geobacteraceae | 2.1 | 0.0002 |
| Pedosphaeraceae | 2.0 | 1.20E-08 |
| Rhodanobacteraceae | 2.0 | 1.20E-08 |
| Chloroflexi | -2.0 | 4.10E-05 |
| Verrucomicrobiaceae | -2.0 | 8.00E-07 |
| Chloroflexi | -2 | 0.0004 |
| Solibacteraceae Subgroup 3 | -2 | 0.0001 |
| Ignavibacteria | -2 | 0.0005 |
| Gammaproteobacteria | -2.1 | 0.0001 |
| Myxococcales | -2.1 | 1.20E-12 |
| Myxococcales | -2.1 | 4.40E-06 |
| Rubritaleaceae | -2.2 | 7.10E-06 |
| Solibacteraceae Subgroup 3 | -2.2 | 0.0005 |
| Pedosphaeraceae | -2.2 | 7.90E-10 |
| Pseudohongiellaceae | -2.2 | 3.30E-07 |
| Microscillaceae | -2.2 | 7.10E-18 |
| Beijerinckiaceae | -2.3 | 0.0004 |
| Woeseiaceae | -2.3 | 1.30E-05 |
| Flavobacteriales | -2.4 | 1.00E-05 |
| Pirellulaceae | -2.6 | 0.0008 |
| Microscillaceae | -2.6 | 0.0005 |
| Phycisphaeraceae | -2.7 | 0.0006 |
| Sandaracinaceae | -2.7 | 0.0008 |
| Chitinophagaceae | -2.7 | 6.50E-09 |
| Chthoniobacteraceae | -2.7 | 4.60E-07 |
| Microscillaceae | -2.7 | 4.10E-08 |
| Demequinaceae | -2.8 | 4.10E-18 |
| Anaerolineae | -2.9 | 1.00E-05 |
| Anaerolineae | -3.0 | 4.50E-05 |
| Solibacteraceae Subgroup 3 | -3.1 | 0.0006 |
| Microscillaceae | -3.3 | 1.20E-08 |
| Planctomycetes | -3 | 0.001 |
| Gammaproteobacteria | -3.4 | 0.0005 |
| Betaproteobacteriales | -3.4 | 4.20E-08 |
| Xanthomonadaceae | -3.4 | 1.80E-10 |
| Flavobacteriales | -3.4 | 0.0008 |
| Chitinophagaceae | -3.5 | 0.0005 |
| Microscillaceae | -3.5 | 1.60E-10 |
| Nannocystaceae | -3 | 0.001 |
| Verrucomicrobiaceae | -3.6 | 0.0001 |
| Anaerolineae | -3.7 | 7.30E-08 |
| Bdellovibrionaceae | -3.7 | 1.30E-05 |
| Hyphomonadaceae | -3.7 | 0.0001 |
| Haliangiaceae | -3.9 | 5.70E-06 |
| Bacillaceae | -3.9 | 0.0008 |
| Rhodanobacteraceae | -3.9 | 1.60E-05 |
| Betaproteobacteriales | -4 | 0.0001 |
| Chthoniobacteraceae | -4 | 0.0003 |
| Phycisphaeraceae | -4 | 0.0005 |
| Pedosphaeraceae | -4.2 | 1.60E-06 |
| Chitinophagaceae | -4.2 | 9.60E-07 |
| Anaerolineae | -4.3 | 1.20E-06 |
| Xanthomonadaceae | -4.3 | 2.20E-09 |
| Gemmatimonadaceae | -4.4 | 1.50E-07 |
| Rhodanobacteraceae | -4.5 | 4.50E-06 |
| Rhodanobacteraceae | -4.5 | 1.10E-07 |
| Rhodanobacteraceae | -4.5 | 1.10E-06 |
| Myxococcales | -4.5 | 7.70E-06 |
| Rhodobacteraceae | -4.6 | 2.20E-06 |
| Microscillaceae | -4.6 | 8.50E-06 |
| Longimicrobiaceae | -4.6 | 2.60E-06 |
| Thermoanaerobacteraceae | -4.6 | 7.50E-06 |
| Myxococcales | -4.9 | 3.60E-09 |
| Rhodanobacteraceae | -4.9 | 2.20E-09 |
| Pirellulaceae | -4.9 | 8.50E-06 |
| Microscillaceae | -5.2 | 1.60E-05 |
| Myxococcales | -5.3 | 7.90E-10 |
| Myxococcales | -7.1 | 7.80E-08 |
| Ctrl vs. CPMS | log_2_ fold change | p adj |
| Xanthomonadaceae | 2.9 | 0.001 |
| Pedosphaeraceae | -2.0 | 8.10E-08 |
| Myxococcales | -2.0 | 1.20E-11 |
| Xanthomonadaceae | -2 | 0.001 |
| Chitinophagaceae | -2.3 | 8.00E-06 |
| Microscillaceae | -2.3 | 4.30E-05 |
| Anaerolineae | -2 | 0.001 |
| Demequinaceae | -2.5 | 3.00E-15 |
| Anaerolineae | -2.9 | 0.0002 |
| Microscillaceae | -3.1 | 8.10E-08 |
| Xanthomonadaceae | -3.1 | 0.0001 |
| Chitinophagaceae | -3 | 0.001 |
| Rhodanobacteraceae | -3.4 | 0.0005 |
| Anaerolineae | -3.5 | 0.0005 |
| Pedosphaeraceae | -3.6 | 0.0003 |
| Myxococcales | -3.7 | 0.0002 |

Table S11. Phospholipid fatty acids (PLFA) calculated per dry mass of soil and soil K_2_SO_4_-extractable C (C_EXT_) and N (N_EXT_) as mg kg^-1^. PLFA_total_, PLFA_bact_, and PLFA_fung_ indicate the PLFA derived from total, bacterial, and fungal biomass, in nmol g^-1^ dry soil. Spri and Aut refers to samples taken at spring and autumn, respectively. CL L and CL U refers to lower and upper 95% confidence interval. Significance of differences between treatment and control (seasonally) is as follows: ***p<0.001, **p<0.01, p<0.05, ^O^p<0.1, ns p>0.1.

| Treat. | Time | PLFA  total | CL L | CL U | PLFA  bact | CL L | CL U | PLFA  fung | CL L | CL U |
| --- | --- | --- | --- | --- | --- | --- | --- | --- | --- | --- |
| FS | Spri | 107.2 | 101.1 | 113.4 | 43.9 | 40.5 | 47.4 | 2.80 | 2.13 | 3.47 |
| LPMS | Spri | 105.6 | 93.1 | 118.1 | 43.8 | 40.4 | 47.3 | 2.72 | 2.05 | 3.39 |
| CPMS | Spri | 100.1 | 89.7 | 110.6 | 41.3 | 37.9 | 44.7 | 2.79 | 2.12 | 3.46 |
| Ctrl | Spri | 105.5 | 95.8 | 115.2 | 42.9 | 39.5 | 46.3 | 2.73 | 2.06 | 3.41 |
| FS | Aut | 98.1 | 86.4 | 109.7 | 40.0 | 35.4 | 44.7 | 3.21 | 2.54 | 3.88 |
| LPMS | Aut | 115.3 | 99.1 | 131.6 | 44.6 | 40.0 | 49.3 | 3.07 | 2.39 | 3.74 |
| CPMS | Aut | 108.3 | 94.2 | 122.4 | 43.8 | 39.2 | 48.5 | 2.62 | 1.95 | 3.30 |
| Ctrl | Aut | 104.4 | 90.9 | 117.9 | 43.0 | 38.3 | 47.6 | 2.75 | 2.08 | 3.42 |
| Treat. | Time | C_EXT_ | CL_L_ | CL_U_ | N_EXT_ | CL_L_ | CL_U_ |  |  |  |
| FS | Spri | 0.029 | 0.024 | 0.035 | 0.008 | 0.006 | 0.010 |  |  |  |
| LPMS | Spri | 0.031 | 0.025 | 0.037 | 0.008 | 0.006 | 0.011 |  |  |  |
| CPMS | Spri | 0.025 | 0.019 | 0.031 | 0.007 | 0.005 | 0.010 |  |  |  |
| Ctrl | Spri | 0.029 | 0.024 | 0.035 | 0.009 | 0.006 | 0.011 |  |  |  |
| FS | Aut | 0.051*** | 0.047 | 0.054 | 0.018 | 0.010 | 0.025 |  |  |  |
| LPMS | Aut | 0.047*** | 0.043 | 0.050 | 0.019 | 0.012 | 0.027 |  |  |  |
| CPMS | Aut | 0.036 | 0.033 | 0.040 | 0.020 | 0.013 | 0.027 |  |  |  |
| Ctrl | Aut | 0.034 | 0.030 | 0.037 | 0.017 | 0.010 | 0.025 |  |  |  |

***Fungal and microbial community composition after organic amendments***

Treatment explained 20% and 18%, and sampling time 7% and 8%, of the variation in community composition (P<0.001) for bacterial and fungal OTUs, respectively. The bacterial and fungal communities in the LPMS treatment differed most from the control, and had the highest pH and microbial N_MB_ and C_MB_ (Fig. 3, Tables S4 and S9). Numerous fungal groups showed significant differences between the treatments and control plots (Table S10). Most distinctively, several members of the Sebacinaceae family showed 3- to 7-fold increases in the treated plots compared with the control. *Tetracladium marchalianum* was more common in the amended plots and e.g., *Mycothermus thermophilus* and *Thermomyces lanuginosus* increased in relative proportion in the LPMS and CPMS plots. Proportion of Sordariales sp. was systematically higher in control plots.

OTUs representing various bacterial groups showed significant differences between the treatments and control plots (Table S11). Bacterial representatives clustering into eight taxa *(Anaerolineae, Chitinophagaceae, Demequinaceae, Microscillaceae, Myxococcales, Pedosphaeraceae, Rhodanobacteraceae and Xanthomonadaceae)* were more common in all amended plots, showing 2-7 fold increases (p adj ≤ 0.0001) compared with the unamended control. FS and LPMS treatments shared 20 bacterial groups that showed higher abundance compared with the control, including e.g., bacterial representatives affiliating to *Bdellovibrionaceae, Beijerinckiaceae, Chthoniobacteraceae, Gemmatimonadaceae, Hyphomonadaceae, Nannocystaceae, Planctomycetes, Rubritalaceae and Verrucomicrobiaceae.* In addition, five bacterial representative groups affiliating to *Azospirillaceae, Blastocatellaceae, Micromonosporaceae, Myxococcaceae and Nitrosomonadaceae* were more common in FS plots, and five different to *Bacillaceae, Chloroflexi, Ignavibacteria, Thermoanaerobacteraceae and Woeseiaceae* in LPMS plots*.*

Several bacteria groups with varying metabolic capabilities increased after all organic amendments (Table S11), e.g., myxobacteria commonly found in soils (Myxococcales) (Dawid 2000), bacteria with roles in cellulose hydrolysis and methanogenesis (Anaerolinea) (Xia et al. 2016), aerobic chemoorganotrophs (Microscillacea) (Hahnke et al. 2016), aerobic organisms with putative β-glucosidase activity (Chitinophagaceae) (Bailey et al. 2013), actinobacteria with numerous functions (Demequinaceae) (Singh et al. 2018), and bacteria isolated from compost (*Arenimonas composti* of the family Xanthomonadaceae) (Jin et al. 2007). However, the bacterial community after FS and LPMS addition was quite similar and consisted of many important or interesting functional groups beneficial to agricultural soils, e.g., parasites for other bacteria (Bdellovibrionaceae) (Starr and Baigent 1966), nitrogen-fixing root-nodule endophyte (Beijerinckiaceae) (De Meyer and Willems 2012), aerobic chemoheterotroph mineralizing organic carbon from plant biomass (Chthoniobacteraceae) (Kant et al. 2011), bacteria adapted to low soil moisture conditions (Gemmatimonadaceae) (DeBruyn et al. 2011), possible producer of novel biologically active compounds (Nannocystaceae) (Dawid 2000), anaerobic ammonia oxidizer (Planctomycetes) (Ward 2013), and aerobic chemoorganotroph from rhizosphere (Verrucomicrobiaceae) (Kielak et al. 2010).

Both FS and LPMS treatment also favored specific bacterial groups. The FS treatment increased beneficial bacteria which, for instance, have the ability to degrade compounds such as chitin, cellulose, lignin, and pectin, playing an important role in the turnover of organic plant material (Micromonosporaceae) (Trujillo et al. 2014), playing major roles in control of the nitrogen cycle by oxidizing ammonia (Nitrosomonadaceae) (Prosser et al. 2014.), and producing diverse secondary compounds with relevance as biocontrol agents (Myxococcaceae) (Dawid 2000). The LPMS treatment for instance induced the appearance of thermophilic and anaerobic bacteria (Bacillaceae, Thermoanaerobacteraceae) (Karakashev et al. 2009, Stackebrandt E. 2014), and lignocellulose-responsive bacteria (Woeseiaceae) (Darjany et al. 2014). It is likely that these anaerobic and thermophilic bacteria originated from processing of the sludge. The higher amount of extractable and microbial biomass C, together with observed changes in bacterial community, may indicate a microbiome that is efficient in storing C in biomass while also releasing labile C from organic compounds, indicating high C use efficiency in soils after organic amendment.

Table S12. Permutational Multivariate Analysis of variance using distance matrices.

|  |  |  | F | R2 | Pr(>F) |
| --- | --- | --- | --- | --- | --- |
| Fungi | ITS2 | time,_1_ | 3.8336 | **0.08** | 0.001 |
|  |  | tr,_3_ | 2.7552 | **0.18** | 0.001 |
|  |  | Residuals,_34_ |  | 0.74 |  |
| Bacteria | 16S | time,_1_ | 3.1808 | **0.07** | 0.001 |
|  |  | tr,_3_ | 3.1322 | **0.20** | 0.001 |
|  |  | Residuals,_35_ |  | 0.74 |  |

**References**

Bailey, V.L., Fansler, S.J., Stegen, J.C. & McCue, L.A. (2013). Linking microbial community structure to β-glucosidic function in soil aggregates. *ISME Journal, 7*, 2044–2053.

Bengtsson-Palme, J., Ryberg, M., Hartmann, M., Branco, S., Godhe, A., De Wit, P...Nilsson, R.H. (2013) Improved software detection and extraction of ITS1 and ITS2 from ribosomal ITS sequences of fungi and other eukaryotes for analysis of environmental sequencing data. *Methods in Ecology and Evolution, 4*, 914–919. doi: 10.1111/2041-210X.12073

Darjany, L.E., Whitcraft, C.R. & Dillon, J.G. (2014). Lignocellulose-responsive bacteria in a southern California salt marsh identified by stable isotope probing. *Frontiers in Microbiology 5, 263*. doi: 10.3389/fmicb.2014.00263

Dawid, W. (2000). Biology and global distribution of myxobacteria in soils. *FEMS Microbiology Reviews 24*, 403-427.

DeBruyn, J., Nixon, L., Fawaz, M., Johnson, M. & Radosevich, M. (2011). Global biogeography and quantitative season dynamics of gemmatimonadetes in soil. *Applied and Environmental Microbiology. 77*, 6295–300. DOI: 10.1128/AEM.05005-11

De Meyer, S.E. & Willems, A. (2012). Multilocus sequence analysis of *Bosea* species and description of *Bosea lupini* sp. nov., *Bosea lathyri* sp. nov. and *Bosea robiniae* sp. nov., isolated from legumes. *International Journal of Systematic and Evolutionary Microbiology, 62*, 2505-2510.

Frostegård, Å., Bååth, E. & Tunlid, A. (1993). Shifts in the structure of soil microbial communities in limed forests as revealed by phospholipid fatty acid analysis. *Soil Biology and Biochemistry, 25*, 723–730.

Fu, L., Niu, B., Zhu, Z., Wu, S. &Li, W. (2012) CD-HIT: accelerated for clustering the next-generation sequencing data. *Bioinformatics, 28*, 3150–3152. doi: 10.1093/bioinformatics/bts565

Hahnke, R.L., Meier-Kolthoff, J.P., García-López, M., Mukherjee, S., Huntemann, M., Ivanova, N.N…Göker, M. (2016). Genome-Based Taxonomic Classification of Bacteroidetes *Frontiers in Microbiology, 7*, 2003. doi: 10.3389/fmicb.2016.02003.

Jin, L., Kim, K.K., Im, W.T., Yang, H.C. & Lee, S.T. (2007). *Aspromonas composti* gen. nov., sp. nov., a novel member of the family Xanthomonadaceae. *International Journal of Systematic and Evolutionary Microbiology, 57*, 1876–80.

Ravi, K., van Passel, M.W.J., Palva, A., Lucas, S., Lapidus, A., del Rio, D.G...Smidt, H. (2011). Genome Sequence of *Chthoniobacter flavus* Ellin428, an Aerobic Heterotrophic Soil Bacterium. *Journal of Bacteriology, 193*, 2902–2903.

Karakashev ,D., Kotay, S.M., Trably, E. & Angelidaki, I. (2009). A strict anaerobic extreme thermophilic hydrogen-producing culture enriched from digested household waste. *Journal of Applied Microbiology, 106*, 1041–1049

Kielak, A., Rodrigues, J.L., Kuramae, E.E., Chain, P.S., van Veen, J.A. & Kowalchuk,G.A. (2010). Phylogenetic and metagenomic analysis of Verrucomicrobia in former agricultural grassland soil. *FEMS Microbiology Ecology, 71*, 23-33.

Kõljalg, U., Nilsson, R.H., Abarenkov, K., Tedersoo, L., Taylor, A.F., Bahram, M...Larsson, K.H. (2013) Towards a unified paradigm for sequence-based identification of fungi. *Molecular Ecology, 22*, 5271–5277. doi: 10.1111/mec.12481

Mahé, F., Rognes, T., Quince, C., de Varqas, C. & Dunthorn, M. (2015). Swarm v2: highly-scalable and high-resolution amplicon clustering. *PeerJ, 3*, e1420–e1420. doi: 10.7717/peerj.1420

Nilsson, R.H., Larsson, K-H., Taylor, A.F.S, Bengtsson-Palme, J., Jeppesen, T.S., Schigel, D., Kennedy, P., Picard, K., Glöckner, F.O., Tedersoo, L., Saar, I., Kõljalg, U. & Abarenkov, K. (2018). The UNITE database for molecular identification of fungi: handling dark taxa and parallel taxonomic classifications. *Nucleic Acids Research, 47*, D259-D264. DOI: 10.1093/nar/gky1022

Pennanen, T., Liski, J., Bååth, E., Kitunen, V., Uotila, J., Westman, C.J. & Fritze, H. (1999). Structure of microbial communities in coniferous forest soils in relation to site fertility and stand development stage. *Microbial Ecology, 38*, 168-179.

Pietikäinen, J. & Fritze, H. (1995). Clear-cutting and prescribed burning in coniferous forest: comparison of effects on soil fungal and total microbial biomass, respiration activity and nitrification. *Soil Biology and Biochemistry, 27*, 101–109.

Prosser J.I., Head I.M. & Stein L.Y. (2014). The Family Nitrosomonadaceae, In Rosenberg E., DeLong E.F., Lory S., Stackebrandt E., Thompson F. (ed.) The Prokaryotes (901-918) (Fourth edition). Springer, Berlin, Heidelberg.

Quast, C., Pruesse, E., Yilmaz, P., Gerken, J., Schweer, T., Yarza, P...Glöckner, F.O. (2013). The SILVA ribosomal RNA gene database project: improved data processing and web-based tools. *Nucleic Acids Research 41*, D590–D596. doi: 10.1093/nar/gks1219

Rognes, T., Flouri, T., Nichols, B., Quince, C. & Mahé, F. (2016) VSEARCH: a versatile open source tool for metagenomics. *PeerJ, 4*, e2584–e2584. doi: 10.7717/peerj.2584

Singh, B.P., Gupta, V.K Passari, A.K. (2018). Actinobacteria: Diversity and Biotechnological Applications: New and Future Developments in Microbial Biotechnology and Bioengineering. Elsevier.

Schloss, P.D., Westcott, S.L., Ryabin, T, Hall, J.R., Hartmann, M., Hollister, E.B...Weber, C.F. (2009) Introducing mothur: Open-Source, Platform-Independent, Community-Supported Software for Describing and Comparing Microbial Communities. *Applied and Environmental Microbiology, 75*, 7537–7541. doi: 10.1128/AEM.01541-09

Stackebrandt, E. (2014). The Family Thermoanaerobacteraceae. In Rosenberg E., DeLong E.F., Lory S., Stackebrandt E. & Thompson F. (ed), The Prokaryotes (413-419). Springer, Berlin, Heidelberg.

[Starr M.P](https://www.ncbi.nlm.nih.gov/pubmed/?term=Starr%20MP%5BAuthor%5D&cauthor=true&cauthor_uid=5327913). & [Baigent N.L](https://www.ncbi.nlm.nih.gov/pubmed/?term=Baigent%20NL%5BAuthor%5D&cauthor=true&cauthor_uid=5327913). (1966). Parasitic interaction of Bdellovibrio Starr MP, Baigent NL. Parasitic interaction of Bdellovibrio bacteriovorus with other bacteria. *Journal of Bacteriology, 91*, 2006–2017.

Trujillo, M.E., Hong, K., Genilloud, O. (2014). The Family Micromonosporaceae. In Rosenberg, E., DeLong, E.F., Lory, S., Stackebrandt E. & Thompson F. (ed), The Prokaryotes (499-569). Springer, Berlin, Heidelberg.

Törmänen, T., Kitunen, V., Lindroos, A-J., Heikkinen, J. & Smolander, A. (2018). How do logging residues of different tree species affect soil N cycling after final felling? *Forest Ecology and Management 427*, 182–189.

Ward, B.B. (2013). Nitrification. In Fath, B. (ed), Encyclopedia of Ecology 351-358 (Second Edition), Elsevier.

Xia, Y., Wang, Y., Wang, Y., Chin, F.Y. & Zhang, T. (2016). Cellular adhesiveness and cellulolytic capacity in Anaerolineae revealed by omics-based genome interpretation. *Biotechnology for Biofuels, 9*, 111.

Yilmaz, P., Parfrey, L.W., Yarza, P., Gerken, J., Pruesse, E., Quast, C….Glöckner, F.O. (2014) The SILVA and “All-species Living Tree Project (LTP)” taxonomic frameworks. *Nucleic Acids Research, 42*, D643–D648. doi: 10.1093/nar/gkt1209
